# The role of age at menarche and age at menopause in Alzheimer’s disease: evidence from a bidirectional mendelian randomization study

**DOI:** 10.18632/aging.203384

**Published:** 2021-08-04

**Authors:** Mingli Li, Jiali Lin, Shuang Liang, Zefeng Chen, Yulan Bai, Xinyang Long, Shengzhu Huang, Zengnan Mo

**Affiliations:** 1 Center for Genomic and Personalized Medicine, Guangxi Medical University, Nanning 530021, Guangxi, China; 2 Guangxi Key Laboratory for Genomic and Personalized Medicine, Nanning 530021, Guangxi, China; 3 Guangxi Collaborative Innovation Center for Genomic and Personalized Medicine, Nanning 530021, Guangxi, China; 4 Guangxi Key Laboratory of Colleges and Universities, Nanning 530021, Guangxi, China; 5 School of Public Health of Guangxi Medical University, Nanning 530021, Guangxi, China; 6 Institute of Urology and Nephrology, First Affiliated Hospital of Guangxi Medical University, Nanning 530021, Guangxi, China

**Keywords:** age at menarche, age at menopause, Alzheimer's disease, mendelian randomization study

## Abstract

The association between endogenous estrogen exposure and Alzheimer’s disease (AD) remains inconclusive in previous observational studies, and few Mendelian randomization (MR) studies have focused on their causality thus far. We performed a bidirectional MR study to clarify the causality and causal direction of age at menarche and age at menopause, which are indicators of endogenous estrogen exposure, on AD risk. We obtained all genetic datasets for the MR analyses using publicly available summary statistics based on individuals of European ancestry from the IEU GWAS database. The MR analyses indicated no significant causal relationship between the genetically determined age at menarche (outlier-adjusted inverse variance weighted odds ratio [IVWOR] = 0.926; 95% confidence interval [CI], 0.803-1.066) or age at menopause (outlier-adjusted IVWOR = 0.981; 95% CI, 0.941-1.022) and AD risk. Similarly, AD did not show any causal association with age at menarche or age at menopause. The sensitivity analyses yielded similar results. In contrast, an inverse association was detected between age at menarche and body mass index (BMI, outlier-adjusted IVW β = -0.043; 95% CI, -0.077 to -0.009). Our bidirectional MR study provides no evidence for a causal relationship between the genetically determined age at menarche or age at menopause and AD susceptibility, or vice versa. However, earlier menarche might be associated with higher adult BMI.

## INTRODUCTION

As the population ages, almost 115.4 million people worldwide will have dementia by 2050, with the main cause being Alzheimer’s disease (AD) [1]. Notably, women have a more than 55% greater lifetime risk of AD at age 65 than men (24.6% vs. 15.5%) and constitute two-thirds of late-onset AD cases [2]. In recent years, sex hormones, especially estrogen [3], have gained increasing attention because accumulating population-based evidence has proposed a protective role for exogenous hormone replacement therapy (HRT) in cognitive decline and dementia progression in postmenopausal females [4, 5]. Because of the lifetime exposure of women to endogenous estrogens, understanding the impact of endocrine event signaling (such as age at menarche and age at menopause) on AD risk is imperative. However, observational findings to date show heterogeneity in the association between endocrine event signaling and the risk of dementia. For example, a large, diverse cohort study showed that delayed menarche increased dementia risk [6], but this association disappeared after adjusting for baseline risk factors of dementia in other studies [7, 8]. Similarly, inconsistent estimates ranged from a modest elevated dementia or AD risk with early onset of natural menopause [9] to an inverse association [6, 10] or an entire loss of statistical evidence [11, 12]. Thus, it is difficult to distinguish whether endogenous estrogen exposure indeed has a causal effect on AD susceptibility or whether this association is completely attributable to other unmeasured potential confounders, such as a high body fat mass or individual socioeconomic factors.

**Figure 1:**
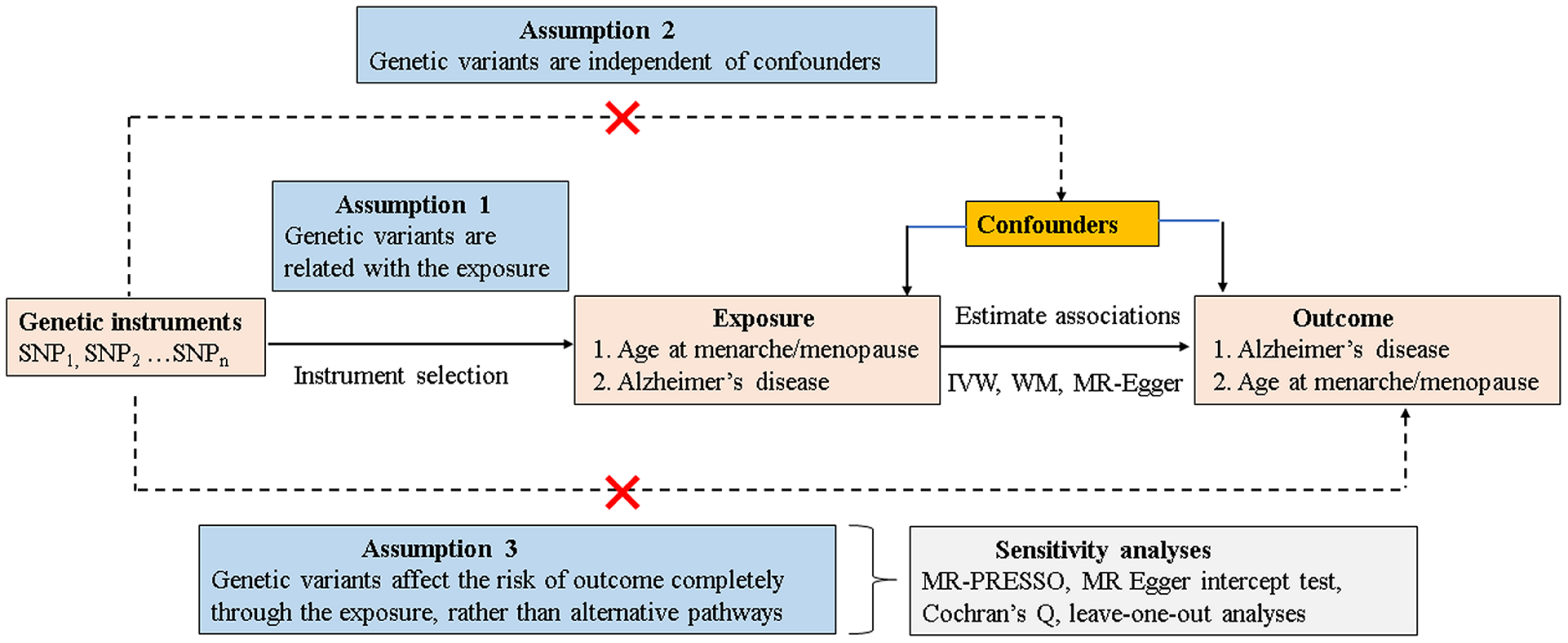
**Schematic model of the MR study.** SNP, single-nucleotide polymorphism; MR, mendelian randomization; IVW, inverse variance–weighted; WM, weighted median.

Mendelian randomization (MR) analysis, using genetic single-nucleotide polymorphisms (SNPs) that are known risk factors of interest as proxy instrument variables (IVs) [13, 14], has been widely established to estimate the causal inference of an exposure on an outcome. As genetic variants are allocated randomly at the time of conception and are relatively independent of environmental and lifestyle factors, the typical confounding factors or reverse causation limited from observational studies could be better mitigated. Nevertheless, MR analysis can provide indirect evidence for a causal association relying on the following three core assumptions [15] (Figure 1): 1) the IVs should be robustly correlated with exposure (assumption 1); 2) the IVs should be independent of any confounders of the exposure-outcome association (assumption 2); and 3) the IVs affect the risk of outcome only through the exposure, rather than any alternative pathways (assumption 3). The latter two assumptions are jointly known as independence from pleiotropy.

The effect of endogenous estrogen exposure on AD risk remains inconclusive in observational studies, and few MR studies have focused on their causal association thus far. Herein, we performed a bidirectional MR study to clarify the causality and causal direction between age at menarche and age at menopause and AD, using publicly available summary statistics from genome-wide association studies (GWAS) based on individuals of European ancestry.

## MATERIALS AND METHODS

We obtained all genetic datasets for the MR analyses using publicly available summary statistics based on individuals of European ancestry from the IEU GWAS database (https://gwas.mrcieu.ac.uk/). Ethical review and informed consent were obtained from the original GWAS. Briefly, in the forward direction, we first analyzed whether genetically determined age at menarche/menopause (SNP exposure) causally affects AD and its relevant traits (SNP outcome), while in the reverse direction, we determined whether genetic predisposition to AD (SNP exposure) affects age at menarche/menopause (SNP outcome).

### IV selection and validation

SNPs associated with exposure at genome-wide significance (P < 5×10^-8^) in the GWAS datasets were selected as IVs. We clumped SNPs to achieve independent loci with a threshold of linkage disequilibrium (LD) r^2^ > 0.001 and a distance of 10,000 kb in PLINK [16]. Then, we extracted the effect estimates of the selected IVs in each outcome GWAS dataset, where target IVs were not available in the outcome of interest. We replaced proxy SNPs in high LD (r^2^ > 0.80) using the online platform LDlink (https://ldlink.nci.nih.gov/). Next, we harmonized the exposure and outcome data using the “TwoSample MR” package to ensure their effects on SNPs corresponding to the same allele or removed all palindromic SNPs from the analysis.

To satisfy the first MR assumption, we applied an F-statistic to evaluate the strength of each selected SNP, and an F-statistic > 10 suggests that the SNP is sufficiently strong to lessen any potential bias [17]. We also computed the variance (R^2^) explained by each IV in the exposure (for F-statistic and R^2^ calculations see Supplementary Methods 1). To address the second MR assumption, we further explored the associations between age at menarche/menopause and the following AD-relevant traits: cognitive performance, body mass index (BMI), smoking behavior and alcohol consumption. To assess the third MR assumption, we performed additional heterogeneity and sensitivity tests to assess the horizontal pleiotropy of the selected SNPs (see section on heterogeneity and sensitivity tests). MR-PRESSO, which assumes that at least 50% of the selected IVs are valid, was further performed to detect and remove any potential pleiotropic outlier SNPs.

### Data sources

**Figure 2:**
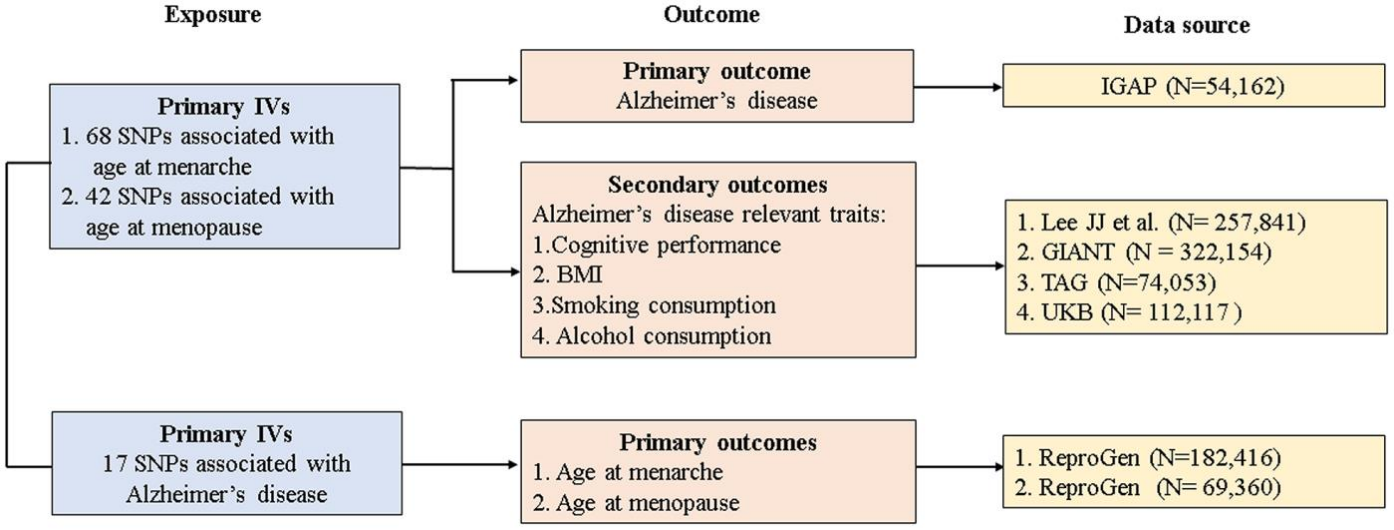
**Study design and data sources.** IGAP, International Genomics of Alzheimer's Project; ReproGen, Reproductive Genetics Consortium; GIANT, Genetic Investigation of Anthropometric Traits; TAG, Tobacco and Genetics Consortium; UKB, UK biobank IVs, instrument variables; SNP, single-nucleotide polymorphism; BMI, body mass index.

The study design and data sources are presented in Figure 2. GWAS summary datasets for age at menarche (n = 182,416) and age at menopause (n = 69,360) were obtained from the Reproductive Genetics (ReproGen) consortium. Briefly, the ReproGen consortium included women with self-reported age at menarche of 9-17 years old and birth year as the only covariates to allow for secular trends in menarche timing [18]. Women with self-reported age at natural menopause of 40-60 years old were included, excluding those with menopause induced by bilateral ovariectomy, hysterectomy, radiation or chemotherapy and those using HRT before menopause [19]. GWAS summary datasets for AD were derived from the largest two-stage study performed by the International Genomics of Alzheimer’s Project (IGAP) [20]. In our MR study, we extracted individual SNPs associated with AD from stage 1 of the IGAP. In stage 1, the IGAP genotyped and imputed data on 7,055,881 SNPs consisting of 17,008 AD cases and 37,154 controls, and adjustments were made for age, sex, and principal components in genetic association analysis.

GWAS summary datasets for AD-relevant traits were selected from the following consortiums or studies: Lee JJ et al. for cognitive performance (n = 257,841) [21], the Genetic Investigation of Anthropometric Traits (GIANT) consortium for BMI (n = 322,154) [22], the Tobacco and Genetics (TAG) consortium for smoking behaviors (n = 74,053) [23], and the UK Biobank (UKB) for alcohol consumption (n = 112,117) [24]. Details of studies and participants are given in Supplementary Methods 2.

### Definition of phenotypes

Menarche was defined as the onset of first menstruation in girls [18]. Menopause was defined as the onset of last naturally occurring menstrual period followed by at least 12 consecutive months of amenorrhea [19]. AD cases were confirmed by autopsy or clinical diagnosis according to the national criteria [20]. Cognitive performance was measured by the respondent’s score on a test of verbal cognition [21]. BMI was calculated as the weight to-squared-height ratio (kg/cm^2^) [22]. Smoking behavior was the average number of cigarettes smoked per day [23]. Alcohol consumption was the average intake in units per week [24].

### Statistical analyses for MR estimates

We estimated an overall causal effect between exposure and outcome using the inverse variance-weighted (IVW) method. IVW is considered the most reliable MR method, assuming all SNPs are valid IVs with no evidence of directional pleiotropy. Given that the results could be biased by the horizontal pleiotropy of IVs, we compared IVW results with other MR methods (i.e., Egger regression and weighted median) whose estimates are more robust to horizontal pleiotropy, although at the expense of lowered statistical power. Egger regression allows for the slope representing the causal effect estimate and the intercept as an indicator of average pleiotropic bias. The weighted median method provides more robust MR estimates; even up to 50%, IVs are invalid. MR-PRESSO was further applied to provide outlier-adjusted estimates, with a significant global test P value < 0.05. Effect estimates are reported in β values where the outcome was continuous (i.e., age at menarche/menopause) and converted to an odds ratio (OR) where the outcome was dichotomous (i.e., AD status).

### Heterogeneity and sensitivity assessment

To further assess the heterogeneities and pleiotropy between IVs, we conducted additional heterogeneity and sensitivity tests. Assuming that all valid IVs have an equivalent effect, Cochran’s Q test was used to estimate the heterogeneities between SNPs. MR Egger intercept regression, representing an indicator of average pleiotropic bias, was conducted to identify the directional pleiotropy between SNPs. Furthermore, “leave-one-out” analyses were performed to estimate the causal effect of outlying IVs by stepwise removing each IV from the MR analysis.

Finally, we searched the potential confounding traits for each IV and their proxies (r^2^ > 0.80) in the PhenoScannerV2 database (http://www.phenoscanner.medschl.cam.ac.uk/) and GWAS catalog (https://www.ebi.ac.uk/gwas/) to stepwise remove the IV with possible pleiotropic effects until Cochran’s Q test made no difference from the null. In the context of age at menarche/menopause-AD, the potential confounders included cognitive performance, BMI, smoking behavior, and alcohol consumption, while in the context of AD age at menarche/menopause, BMI was most likely to be a major confounder.

All analyses were conducted using R statistical software (version 4.0.2) with the R packages “TwoSample MR” and “MR-PRESSO,” and P < 0.05 was considered statistically significant.

### Sample size and power calculations

We estimated MR power for binary and continuous outcomes at a two-sided α of 0.05 using the mRnd power calculation tool (https://shiny.cnsgenomics.com/mRnd/). Both forward- and reverse-direction MR analyses had sufficient (> 80%) power to detect a statistically significant effect, suggesting that the associations did not arise from chance. Furthermore, all sample sizes of the corresponding GWAS summary datasets were much larger than the sample size required for 80% power. Sample size and power calculations are given in Supplementary Methods 3 and Supplementary Table 1.

## RESULTS

### IV selection

In the forward direction, 68 SNPs associated with age at menarche were included as IVs and together accounted for 3.55% of the total variance. Meanwhile, 42 SNPs associated with age at menopause were eligible as IVs and together accounted for 4.69% of the total variance. In the reverse direction, 17 SNPs associated with AD were selected as IVs and together accounted for 3.37% of the total variance. The F-statistic value for each selected IV was more than 10, suggesting that the selected SNPs were sufficiently strong and that the causal estimate was unlikely to be biased by weak IVs. The association between each SNP exposure and the corresponding SNP outcome is presented in Supplementary Tables 2–4.

### MR estimates of age at menarche and AD

As presented in Table 1, after removing 14 SNPs for being palindromic, no causal association was observed between the genetically determined age at menarche and AD across the three MR methods for 54 SNPs (all P > 0.05). Meanwhile, MR-PRESSO did not detect any potential outliers (global P = 0.251); Cochran’s Q statistics showed no notable heterogeneities between IVs (Q_IVW_ = 63.114, P_IVW_ = 0.161; Q_MR-Egger_ = 62.143, P_MR-Egger_ = 0.158); and no horizontal pleiotropy (intercept = 0.009; P = 0.372) was observed in the MR Egger intercept test (Table 2). However, “leave-one-out” analyses indicated that the causal estimate of IVW was driven by four SNPs (i.e., rs1659127, rs2947411, rs740077 and rs6747380) (Supplementary Figure 1A). Therefore, we searched the PhenoScanner database and mapped SNPs to known genes implicated in the GWAS catalog to identify those nominally associated with AD or its relevant traits (Supplementary Table 5). Finally, 23 of the 54 SNPs were removed for being potentially pleiotropic, and the MR estimate remained null after removing the outliers (outlier-adjusted IVWOR, 0.926 for AD per 1-SD increase in mean age at menarche; 95% CI, 0.803-1.066, P = 0.284). The weighted median and MR Egger analysis yielded a similar pattern of effects (Table 1), with no single SNP driving the results (Supplementary Figure 1B).

**Table 1:** MR results for the relationships between age at menarche/menopause and AD.

**Exposure-outcome**	**Method**		**OR(95%CI)^a^**	***P* value**	**No. of SNPs**
Age at menarche-AD	Main model ^b^	IVW	0.926 (0.803-1.066)	0.284	31
		Weighted median	0.972 (0.801-1.179)	0.770	31
		MR Egger	1.160 (0.639-2.107)	0.629	31
	With outliers ^c^	IVW	0.903 (0.807-1.010)	0.075	54
		Weighted median	0.939 (0.800-1.102)	0.444	54
		MR Egger	0.749 (0.491-1.142)	0.185	54
Age at menarche-AD	Main model ^b^	IVW	0.975 (0.935-1.017)	0.241	23
		Weighted median	0.985 (0.931-1.043)	0.612	23
		MR Egger	0.939 (0.860-1.025)	0.172	23
	With outliers ^c^	IVW	0.991 (0.957-1.026)	0.611	38
		Weighted median	0.985 (0.941-1.031)	0.520	38
		MR Egger	0.954 (0.883-1.032)	0.251	38

Abbreviations: MR, Mendelian randomization; AD, Alzheimer’s disease; IVW, inverse variance-weighted; OR, odds ratio; SNP, single-nucleotide polymorphism.

^a^Indicates odds ratio for AD per 1-SD increase in mean age at menarche/menopause.

^b^Indicates model removal of potential pleiotropic IVs.

^c^Indicates model without removal of potential pleiotropic IVs.

**Table 2:** The heterogeneity and sensitivity results of age at menarche/menopause and AD before and after removal of pleiotropic IVs.

**Exposure-outcome**	**No. of SNPs**		**MR-PRESSO**		**MR Egger intercept**			**Cochran's heterogeneity test**			
			**Global *P* value**		**Intercept value**	***P* value**		**IVW-Q value**	**IVW-*P* value**	**Egger-Q value**	**Egger-*P* value**
Age at menarche-AD	54^a^		0.251		0.009	0.372		63.114	0.161	62.143	0.158
Age at menarche-AD	31^b^		0.824		-0.010	0.451		32.074	0.364	31.440	0.345
Age at menopause-AD	38^a^		0.052		0.008	0.300		54.193	0.034	52.581	0.037
Age at menopause-AD	23^b^		0.226		0.008	0.342		26.473	0.189	27.664	0.187
AD-age at menarche	6		0.489		-0.003	0.850		4.319	0.504	4.276	0.370
AD-age at menopause	6		0.052		-0.058	0.266		4.222	0.518	2.549	0.636

Abbreviations: MR, Mendelian randomization; AD, Alzheimer’s disease; IVW, inverse variance-weighted; OR, odds ratio; SNP, single-nucleotide polymorphism.

^a^Indicates model removal of potential pleiotropic IVs.

^b^Indicates model without removal of potential pleiotropic IVs.

In the reverse direction, only six of the 17 IVs were found in the age at menarche summary datasets and were included for MR analyses. We discovered no statistically significant association between genetic predisposition to AD and age at menarche (IVWβ = 0.006 in mean age at menarche per AD vs. control status; 95% CI, -0.039 to 0.051, P = 0.793). The weighted median and MR Egger analyses yielded a similar pattern of effects, and no potential outliers, notable heterogeneities, or horizontal pleiotropy were detected (Tables 2, 3), without a single SNP driving the results (Supplementary Figure 2). Neither the PhenoScanner database nor the GWAS catalog detected the IVs associated with BMI (Supplementary Figure 2 and Supplementary Table 6).

**Table 3:** MR results for the relationships between AD and age at menarche/menopause.

**Exposure-outcome**	**Method**	B(95%CI)*	***P* value**	**No. of SNPs**
AD-age at menarche	IVW	0.006 (-0.039 to 0.051)	0.793	6
	Weighted median	0.000 (-0.063 to 0.063)	0.990	6
	MR Egger	0.029 (-0.195 to 0.252)	0.815	6
AD-age at menopause	IVW	-0.044 (-0.206 to 0.119)	0.598	6
	Weighted median	-0.114 (-0.332 to 0.104)	0.306	6
	MR Egger	0.374 (-0.280 to 1.028)	0.325	6

Abbreviations: MR, mendelian randomization; AD, Alzheimer’s disease; IVW, inverse variance-weighted; OR, odds ratio; SNP, single-nucleotide polymorphism.

*Indicates change in mean year at menarche/menopause per AD vs control status.

### MR estimates of age at menopause and AD

Similarly, we also found no evidence of a causal relationship between the genetically determined age at menopause and AD, regardless of whether pleiotropic SNPs were removed (for 24 SNPs, outlier-adjusted IVWOR, 0.981 for AD per 1-SD increase in mean age at menopause; 95% CI, 0.941-1.022, P = 0.352; for 38 SNPs, IVWOR, 0.991 for AD per 1-SD increase in mean age at menopause; 95% CI, 0.957-1.026, P = 0.611); or between genetic predisposition to AD and age at menopause (for six SNPs, IVWβ = -0.044 in mean age at menopause per AD vs. control status; 95% CI, -0.206 to 0.119, P=0.598). A similar pattern of effects was also indicated in the weighted median and MR Egger analyses (Tables 1–3, Supplementary Tables 6, 7 and Supplementary Figures 2, 3).

### MR estimates of age at menarche/menopause and AD-relevant traits

**Figure 3:**
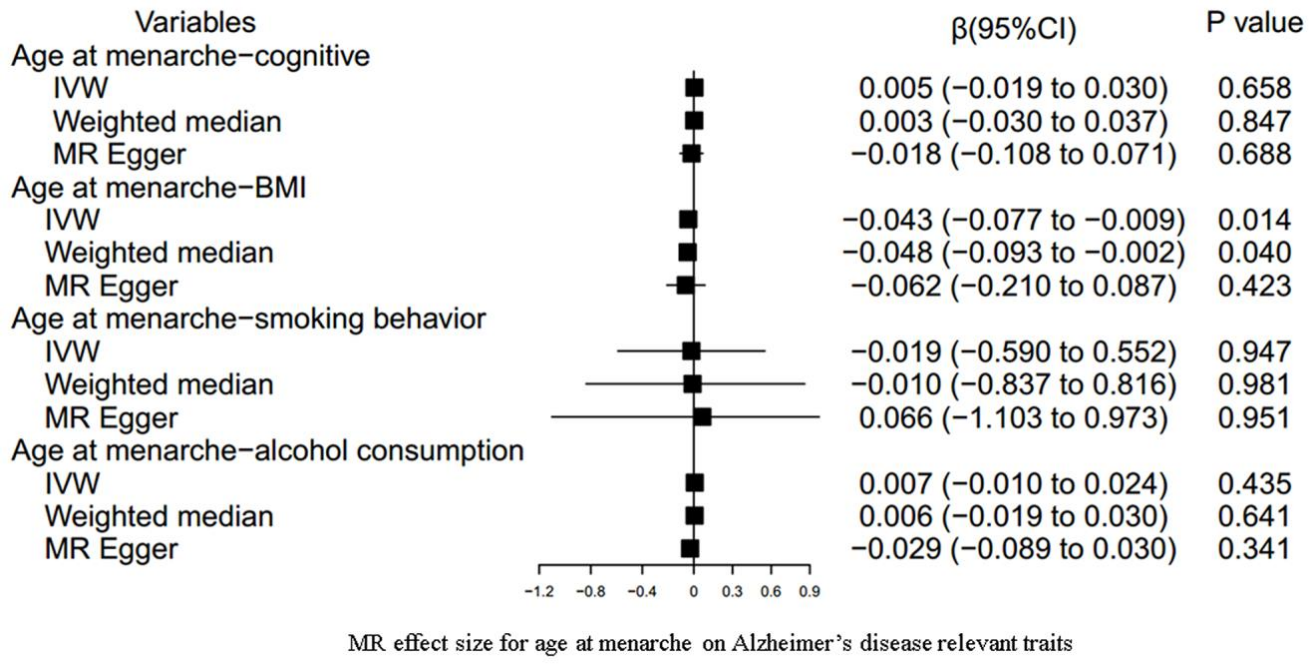
**MR estimate plot for age at menarche on Alzheimer’s disease relevant traits.** IVW indicates inverse variance–weighted method.

**Figure 4:**
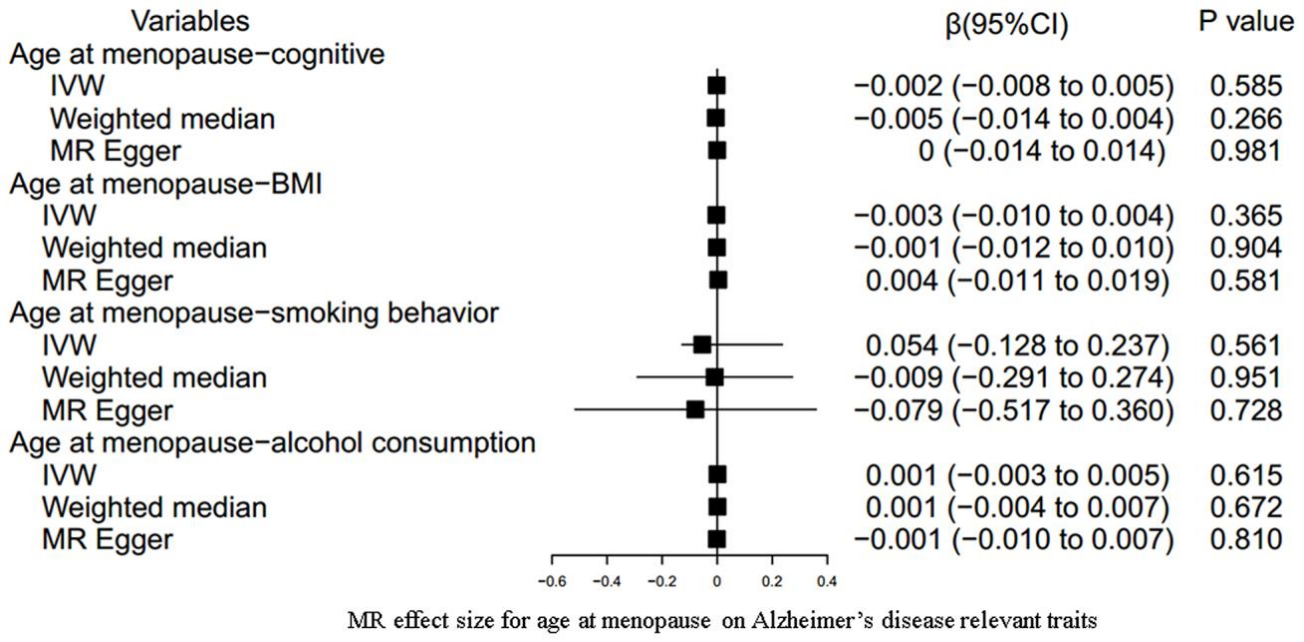
**MR estimate plot for age at menopause on Alzheimer’s disease relevant traits.** IVW indicates inverse variance–weighted method.

Our results indicated an inverse association between age at menarche and BMI (for 31 SNPs, outlier-adjusted IVW β = -0.043; 95% CI, -0.077 to -0.009, P = 0.014; outlier-adjusted weighted median β = -0.048; 95% CI, -0.093 to -0.002, P = 0.040), although the MR Egger regression analysis suggested a null causal effect. However, neither age at menarche nor age at menopause had a significant association with the other remaining AD-relevant traits across the three MR methods (all P > 0.05) (Figures 3, 4 and Supplementary Table 8).

## DISCUSSION

In this large bidirectional MR study, we did not discover a causal relationship between the genetically determined age at menarche or age at menopause on AD susceptibility, or vice versa. Additionally, multiple heterogeneity and sensitivity analyses have been performed to detect and remove any potential of pleiotropy (i.e., where the genetic IVs do not have direct effects on outcomes independent of exposures), making these results more reliable and transparent.

Age at menarche, as a high polygenetic childhood trait, is a prominent milestone of puberty timing in women [18]. MR evidence has suggested the detrimental effects of early menarche on diverse health outcomes including obesity [25], cardiovascular disease [26], cancer [27], and all-cause mortality [28]. Our MR findings corroborated the results from some prospective studies [8, 29] showing a null association between self-reported age at menarche and AD risk, although some studies [30, 31] found a positive association. For example, X Hong et al. [30] reported that increased AD risk paralleled an increased age at menarche (adjusted OR = 1.16 for each increased year, P = 0.0342). Gilsanz et al. [31] found a hazard ratio (HR) of 1.23 (95% CI, 1.01-1.50) for age at menarche (≥ 16 vs.13.0 years) in association with dementia, independent of demographics and life course health indicators. These conflicting findings observed in conventional observational studies are possibly due to reverse causation bias or improper adjustment for residual confounders that underlie the causal pathway, such as childhood or adult obesity.

Higher BMI in childhood is linked with earlier menarche [32], and increases AD risk [33]. In our MR study, both the IVW method and weighted median methods consistently demonstrated an inverse association between age at menarche and BMI after removing their high degree of genetic overlap SNPs or SNPs associated with childhood BMI, although MR Egger regression analysis yielded a null causal effect. In fact, the first two MR methods have better accuracy in the causal estimates [15], greater empirical power [34], and better finite-sample type I error rates [15] compared to MR Egger regression. Thus, it is reasonable to believe that earlier menarche could causally influence a higher risk for BMI in adulthood, in line with the results from previous MR studies [35, 36]. Namely, females who have earlier menarche onset are more likely to develop adiposity, implying that BMI might be a critical potential confounder of the age at menarche-AD association in observational studies. Nevertheless, some studies also argued that age at menarche has a limited influence on future adiposity because higher adiposity in childhood could induce earlier puberty [37] and then track forward into adulthood [38, 39]. Therefore, a more extensive sample size and more rigorously designed studies are necessary to resolve their causal direction. Contrary to other epidemiological evidence [40–42], our results indicate no causal associations between the age at menarche and other relevant traits, such as cognitive performance, smoking behavior, and alcohol consumption.

Menopause marks reproductive senescence and is highly heritable with estimates of 0.40-0.70 from twin and sibling studies [43, 44]. Menopausal women are susceptible to lung function [45], osteoporosis [46], cardiovascular disease [46], and age-related morbidity and mortality outcomes [47]. Evidence from accumulating neurobiological studies [48, 49] has declared that later natural menopause delays cognitive decline or AD risk after full adjustment, pointing out the early endocrine aging process as the optimal window for preventing or delaying progression of AD in women. Later natural menopause onset is likely to involve estrogen receptor β function, which regulates brain-derived neurotrophic factors and in turn solidifies memory formation and storage [50]. However, in a 44-year longitudinal population study of Swedish women with natural menopause, AD risk increased as the age at menopause increased (adjusted OR = 1.07, 95% CI, 1.02-1.12) [29]. Compared to women who experienced menopause at younger than 48 years of age, the adjusted rate ratio (RR) for women aged 50-52 years was 1.64 (95% CI, 1.05-2.56), but there was no association with women older than 52 years of age (adjusted RR = 1.47, 95% CI, 0.88-2.46) [7]. Interestingly, our MR study detected no significant causal relationship between the genetically determined age at menopause and AD, consistent with a population-based cohort study [8]. The disparities in these observational findings could be explained by not fully ruling out some possible confounders. For example, HRT use, which has been proposed for cognitive improvement or AD treatment [5], or the APOE locus, which is a major genetic risk factor for AD [51], are possible confounders. In contrast, in our MR study, all the women included from the GWAS datasets associated with age at menopause had experienced natural menopause, excluding those induced by using HRT, surgery or radiation before menopause. Furthermore, the SNPs associated with age at menopause linked to the APOE locus were removed to minimize type I error. Thus, it is believed that the interpretation of our MR results may be more credible. In addition, our study also indicated that there were no causal effects of age at menopause on any AD-relevant trait, although some previous observational studies have supported possible links [48, 52–54].

In the reverse direction, our findings also suggested a nonsignificant association between genetic predisposition to AD and the age at menarche/menopause. That is, neither of these two biological traits is a consequence nor the cause of AD, although the beneficial effects of estrogens on the central nervous system are biologically plausible. Underlying mechanisms decrease the toxicities of amyloid-beta (Aβ) and glutamate [55], diminish tau protein hyperphosphorylation [56], reduce inflammation and improve synaptic plasticity in the brain [57]. Menarche and menopause currently show considerable variability between women with a high prevalence of obesity, especially in the age of natural menopause onset [58]. In addition, the absolute amounts of estrogens and mechanisms of endogenous estrogens are different from those of exogenous estrogens. We thus need to better understand the impact of prolonged exposure to endogenous estrogens on dementia or AD risk, rather than blindly applying HRT. The “time hypothesis” theory suggests that estrogens exert dual effects of being neuroprotective for healthy cells but neurotoxic in diseased cells [59]. This theory could partially explain why earlier menarche does not affect cognition. Meanwhile, HRT has a limited positive influence on dementia risk when administered within five years of menopause but causes subsequent adverse effects [4, 60]. Overall, the implications of our findings are that the genetically determined onset of menarche and menopause has limited beneficial effects on AD risk. Efforts to supplement estrogens as an effective prevention measure for AD are worthy of further verification.

To our knowledge, this is the first bidirectional MR study focused on the causality and causal direction between age at menarche/menopause and AD. The strengths of this study include the large sample size from GWAS summary datasets, and the robustness of the inherent confounding factors or reverse causation from the observational studies. Our study also has some limitations. First, MR is a reliable way to assess causality in the absence of pleiotropy. There is high risk of pleiotropy in MR analyses because many selected SNPs have diverse or uncertain biological functions. To address this, we attempted to perform multiple sensitivity tests to thoroughly examine pleiotropic effects. It is reassuring that the MR estimates were robust, indicating negligible bias from other apparent sources of pleiotropy. Second, to minimize population stratification bias, our analyses were restricted to individuals of European ancestry and might not be generalizable to non-Europeans. Thus, evidence on the shared genetic variants for the age at menarche/menopause or AD across ethnicities needs to be further validated. Third, since the age at menarche and menopause are based on self-reported information, potential recall bias and measurement error may reduce statistical power to some extent. Last, our analyses only included genetic datasets for the target phenotypes because their individual epidemiological datasets were not publicly accessible. Hence, we could not explore the causal effect of the reproductive period (i.e., technically defined by the time from menarche to menopause) on AD risk. Fortunately, some important confounders, such as age and sex, were well adjusted in the corresponding original GWAS, which may partially lower confounding bias. Moreover, evidence for a high correlation coefficient of 0.93 between age at menopause and the reproductive period was supported by a recent MR study [45], which indirectly indicated a causal effect of the reproductive period on AD risk in our MR analyses.

In conclusion, our bidirectional MR study provided no evidence for a causal effect of the genetically determined age at menarche or age at menopause on AD susceptibility, or vice versa. In contrast, earlier menarche might be associated with higher adult BMI. Further studies combining individual epidemiological and genetic data are warranted to validate and replicate these findings.

## Supplementary Material

Supplementary Methods

Supplementary Figures

Supplementary Tables

## References

[1] Prince M, Bryce R, Albanese E, Wimo A, Ribeiro W, Ferri CP. The global prevalence of dementia: a systematic review and metaanalysis. Alzheimers Dement. 2013; 9:63–75.e2. 10.1016/j.jalz.2012.11.007 23305823

[2] Chêne G, Beiser A, Au R, Preis SR, Wolf PA, Dufouil C, Seshadri S. Gender and incidence of dementia in the Framingham Heart Study from mid-adult life. Alzheimers Dement. 2015; 11:310–20. 10.1016/j.jalz.2013.10.005 24418058 PMC4092061

[3] Lee JH, Jiang Y, Han DH, Shin SK, Choi WH, Lee MJ. Targeting estrogen receptors for the treatment of Alzheimer’s disease. Mol Neurobiol. 2014; 49:39–49. 10.1007/s12035-013-8484-9 23771838

[4] Whitmer RA, Quesenberry CP, Zhou J, Yaffe K. Timing of hormone therapy and dementia: the critical window theory revisited. Ann Neurol. 2011; 69:163–69. 10.1002/ana.22239 21280086 PMC3058824

[5] Song YJ, Li SR, Li XW, Chen X, Wei ZX, Liu QS, Cheng Y. The Effect of Estrogen Replacement Therapy on Alzheimer’s Disease and Parkinson’s Disease in Postmenopausal Women: A Meta-Analysis. Front Neurosci. 2020; 14:157. 10.3389/fnins.2020.00157 32210745 PMC7076111

[6] Yoo JE, Shin DW, Han K, Kim D, Won HS, Lee J, Kim SY, Nam GE, Park HS. Female reproductive factors and the risk of dementia: a nationwide cohort study. Eur J Neurol. 2020; 27:1448–58. 10.1111/ene.14315 32396982

[7] Geerlings MI, Ruitenberg A, Witteman JC, van Swieten JC, Hofman A, van Duijn CM, Breteler MM, Launer LJ. Reproductive period and risk of dementia in postmenopausal women. JAMA. 2001; 285:1475–81. 10.1001/jama.285.11.1475 11255424

[8] Prince MJ, Acosta D, Guerra M, Huang Y, Jimenez-Velazquez IZ, Llibre Rodriguez JJ, Salas A, Sosa AL, Chua KC, Dewey ME, Liu Z, Mayston R, Valhuerdi A. Reproductive period, endogenous estrogen exposure and dementia incidence among women in Latin America and China; A 10/66 population-based cohort study. PLoS One. 2018; 13:e0192889. 10.1371/journal.pone.0192889 29489847 PMC5831083

[9] Coppus AM, Evenhuis HM, Verberne GJ, Visser FE, Eikelenboom P, van Gool WA, Janssens AC, van Duijn CM. Early age at menopause is associated with increased risk of dementia and mortality in women with Down syndrome. J Alzheimers Dis. 2010; 19:545–50. 10.3233/JAD-2010-1247 20110600

[10] Mosconi L, Rahman A, Diaz I, Wu X, Scheyer O, Hristov HW, Vallabhajosula S, Isaacson RS, de Leon MJ, Brinton RD. Increased Alzheimer’s risk during the menopause transition: A 3-year longitudinal brain imaging study. PLoS One. 2018; 13:e0207885. 10.1371/journal.pone.0207885 30540774 PMC6291073

[11] Bove R, Secor E, Chibnik LB, Barnes LL, Schneider JA, Bennett DA, De Jager PL. Age at surgical menopause influences cognitive decline and Alzheimer pathology in older women. Neurology. 2014; 82:222–29. 10.1212/WNL.0000000000000033 24336141 PMC3902759

[12] Georgakis MK, Kalogirou EI, Diamantaras AA, Daskalopoulou SS, Munro CA, Lyketsos CG, Skalkidou A, Petridou ET. Age at menopause and duration of reproductive period in association with dementia and cognitive function: A systematic review and meta-analysis. Psychoneuroendocrinology. 2016; 73:224–43. 10.1016/j.psyneuen.2016.08.003 27543884

[13] Evans DM, Davey Smith G. Mendelian Randomization: New Applications in the Coming Age of Hypothesis-Free Causality. Annu Rev Genomics Hum Genet. 2015; 16:327–50. 10.1146/annurev-genom-090314-050016 25939054

[14] Smith GD, Ebrahim S. ‘Mendelian randomization’: can genetic epidemiology contribute to understanding environmental determinants of disease? Int J Epidemiol. 2003; 32:1–22. 10.1093/ije/dyg070 12689998

[15] Bowden J, Davey Smith G, Haycock PC, Burgess S. Consistent Estimation in Mendelian Randomization with Some Invalid Instruments Using a Weighted Median Estimator. Genet Epidemiol. 2016; 40:304–14. 10.1002/gepi.21965 27061298 PMC4849733

[16] Purcell S, Neale B, Todd-Brown K, Thomas L, Ferreira MA, Bender D, Maller J, Sklar P, de Bakker PI, Daly MJ, Sham PC. PLINK: a tool set for whole-genome association and population-based linkage analyses. Am J Hum Genet. 2007; 81:559–75. 10.1086/519795 17701901 PMC1950838

[17] Palmer TM, Lawlor DA, Harbord RM, Sheehan NA, Tobias JH, Timpson NJ, Davey Smith G, Sterne JA. Using multiple genetic variants as instrumental variables for modifiable risk factors. Stat Methods Med Res. 2012; 21:223–42. 10.1177/0962280210394459 21216802 PMC3917707

[18] Perry JR, Day F, Elks CE, Sulem P, Thompson DJ, Ferreira T, He C, Chasman DI, Esko T, Thorleifsson G, Albrecht E, Ang WQ, Corre T, et al, and Australian Ovarian Cancer Study, and GENICA Network, and kConFab, and LifeLines Cohort Study, and InterAct Consortium, and Early Growth Genetics (EGG) Consortium. Parent-of-origin-specific allelic associations among 106 genomic loci for age at menarche. Nature. 2014; 514:92–97. 10.1038/nature13545 25231870 PMC4185210

[19] Day FR, Ruth KS, Thompson DJ, Lunetta KL, Pervjakova N, Chasman DI, Stolk L, Finucane HK, Sulem P, Bulik-Sullivan B, Esko T, Johnson AD, Elks CE, et al, and PRACTICAL consortium, and kConFab Investigators, and AOCS Investigators, and Generation Scotland, and EPIC-InterAct Consortium, and LifeLines Cohort Study. Large-scale genomic analyses link reproductive aging to hypothalamic signaling, breast cancer susceptibility and BRCA1-mediated DNA repair. Nat Genet. 2015; 47:1294–303. 10.1038/ng.3412 26414677 PMC4661791

[20] Lambert JC, Ibrahim-Verbaas CA, Harold D, Naj AC, Sims R, Bellenguez C, DeStafano AL, Bis JC, Beecham GW, Grenier-Boley B, Russo G, Thorton-Wells TA, Jones N, et al, and European Alzheimer’s Disease Initiative (EADI), Genetic and Environmental Risk in Alzheimer’s Disease, Alzheimer’s Disease Genetic Consortium, and Cohorts for Heart and Aging Research in Genomic Epidemiology. Meta-analysis of 74,046 individuals identifies 11 new susceptibility loci for Alzheimer’s disease. Nat Genet. 2013; 45:1452–58. 10.1038/ng.2802 24162737 PMC3896259

[21] Lee JJ, Wedow R, Okbay A, Kong E, Maghzian O, Zacher M, Nguyen-Viet TA, Bowers P, Sidorenko J, Karlsson Linnér R, Fontana MA, Kundu T, Lee C, et al, and 23andMe Research Team, COGENT (Cognitive Genomics Consortium), and Social Science Genetic Association Consortium. Gene discovery and polygenic prediction from a genome-wide association study of educational attainment in 1.1 million individuals. Nat Genet. 2018; 50:1112–21. 10.1038/s41588-018-0147-3 30038396 PMC6393768

[22] Locke AE, Kahali B, Berndt SI, Justice AE, Pers TH, Day FR, Powell C, Vedantam S, Buchkovich ML, Yang J, Croteau-Chonka DC, Esko T, Fall T, et al, and LifeLines Cohort Study, and ADIPOGen Consortium, and AGEN-BMI Working Group, and CARDIOGRAMplusC4D Consortium, and CKDGen Consortium, and GLGC, and ICBP, and MAGIC Investigators, and MuTHER Consortium, and MIGen Consortium, and PAGE Consortium, and ReproGen Consortium, and GENIE Consortium, and International Endogene Consortium. Genetic studies of body mass index yield new insights for obesity biology. Nature. 2015; 518:197–206. 10.1038/nature14177 25673413 PMC4382211

[23] Tobacco and Genetics Consortium. Genome-wide meta-analyses identify multiple loci associated with smoking behavior. Nat Genet. 2010; 42:441–47. 10.1038/ng.571 20418890 PMC2914600

[24] Clarke TK, Adams MJ, Davies G, Howard DM, Hall LS, Padmanabhan S, Murray AD, Smith BH, Campbell A, Hayward C, Porteous DJ, Deary IJ, McIntosh AM. Genome-wide association study of alcohol consumption and genetic overlap with other health-related traits in UK Biobank (N=112 117). Mol Psychiatry. 2017; 22:1376–84. 10.1038/mp.2017.153 28937693 PMC5622124

[25] Widén E, Silventoinen K, Sovio U, Ripatti S, Cousminer DL, Hartikainen AL, Laitinen J, Pouta A, Kaprio J, Järvelin MR, Peltonen L, Palotie A. Pubertal timing and growth influences cardiometabolic risk factors in adult males and females. Diabetes Care. 2012; 35:850–56. 10.2337/dc11-1365 22338106 PMC3308310

[26] Canoy D, Beral V, Balkwill A, Wright FL, Kroll ME, Reeves GK, Green J, Cairns BJ, and Million Women Study Collaborators*. Age at menarche and risks of coronary heart and other vascular diseases in a large UK cohort. Circulation. 2015; 131:237–44. 10.1161/CIRCULATIONAHA.114.010070 25512444

[27] Day FR, Thompson DJ, Helgason H, Chasman DI, Finucane H, Sulem P, Ruth KS, Whalen S, Sarkar AK, Albrecht E, Altmaier E, Amini M, Barbieri CM, et al, and LifeLines Cohort Study, and InterAct Consortium, and kConFab/AOCS Investigators, and Endometrial Cancer Association Consortium, and Ovarian Cancer Association Consortium, and PRACTICAL consortium. Genomic analyses identify hundreds of variants associated with age at menarche and support a role for puberty timing in cancer risk. Nat Genet. 2017; 49:834–41. 10.1038/ng.3841 28436984 PMC5841952

[28] Charalampopoulos D, McLoughlin A, Elks CE, Ong KK. Age at menarche and risks of all-cause and cardiovascular death: a systematic review and meta-analysis. Am J Epidemiol. 2014; 180:29–40. 10.1093/aje/kwu113 24920784 PMC4070937

[29] Najar J, Östling S, Waern M, Zettergren A, Kern S, Wetterberg H, Hällström T, Skoog I. Reproductive period and dementia: A 44-year longitudinal population study of Swedish women. Alzheimers Dement. 2020; 16:1153–63. 10.1002/alz.12118 32573980

[30] Hong X, Zhang X, Li H. [A case-control study of endogenous estrogen and risk of Alzheimer’s disease]. Zhonghua Liu Xing Bing Xue Za Zhi. 2001; 22:379–82. 11769698

[31] Gilsanz P, Lee C, Corrada MM, Kawas CH, Quesenberry CP Jr, Whitmer RA. Reproductive period and risk of dementia in a diverse cohort of health care members. Neurology. 2019; 92:e2005–14. 10.1212/WNL.0000000000007326 30923235 PMC6511081

[32] Davison KK, Susman EJ, Birch LL. Percent body fat at age 5 predicts earlier pubertal development among girls at age 9. Pediatrics. 2003; 111:815–21. 10.1542/peds.111.4.815 12671118 PMC2530923

[33] Beydoun MA, Beydoun HA, Wang Y. Obesity and central obesity as risk factors for incident dementia and its subtypes: a systematic review and meta-analysis. Obes Rev. 2008; 9:204–18. 10.1111/j.1467-789X.2008.00473.x 18331422 PMC4887143

[34] Burgess S, Butterworth A, Thompson SG. Mendelian randomization analysis with multiple genetic variants using summarized data. Genet Epidemiol. 2013; 37:658–65. 10.1002/gepi.21758 24114802 PMC4377079

[35] Bell JA, Carslake D, Wade KH, Richmond RC, Langdon RJ, Vincent EE, Holmes MV, Timpson NJ, Davey Smith G. Influence of puberty timing on adiposity and cardiometabolic traits: A Mendelian randomisation study. PLoS Med. 2018; 15:e1002641. 10.1371/journal.pmed.1002641 30153260 PMC6112630

[36] Gill D, Brewer CF, Del Greco MF, Sivakumaran P, Bowden J, Sheehan NA, Minelli C. Age at menarche and adult body mass index: a Mendelian randomization study. Int J Obes (Lond). 2018; 42:1574–81. 10.1038/s41366-018-0048-7 29549348

[37] Ahmed ML, Ong KK, Dunger DB. Childhood obesity and the timing of puberty. Trends Endocrinol Metab. 2009; 20:237–42. 10.1016/j.tem.2009.02.004 19541497

[38] Johnson W, Li L, Kuh D, Hardy R. How Has the Age-Related Process of Overweight or Obesity Development Changed over Time? Co-ordinated Analyses of Individual Participant Data from Five United Kingdom Birth Cohorts. PLoS Med. 2015; 12:e1001828. 10.1371/journal.pmed.1001828 25993005 PMC4437909

[39] Wade KH, Chiesa ST, Hughes AD, Chaturvedi N, Charakida M, Rapala A, Muthurangu V, Khan T, Finer N, Sattar N, Howe LD, Fraser A, Lawlor DA, et al. Assessing the causal role of body mass index on cardiovascular health in young adults: Mendelian randomization and recall-by-genotype analyses. Circulation. 2018; 138:2187–201. 10.1161/CIRCULATIONAHA.117.033278 30524135 PMC6250296

[40] Verhoef M, van den Eijnden RJ, Koning IM, Vollebergh WA. Age at menarche and adolescent alcohol use. J Youth Adolesc. 2014; 43:1333–45. 10.1007/s10964-013-0075-6 24327296

[41] Chen Y, Liu Q, Li W, Deng X, Yang B, Huang X. Association of prenatal and childhood environment smoking exposure with puberty timing: a systematic review and meta-analysis. Environ Health Prev Med. 2018; 23:33. 10.1186/s12199-018-0722-3 30021511 PMC6052528

[42] Chou HT, Wu PY, Huang JC, Chen SC, Ho WY. Late Menarche, Not Reproductive Period, Is Associated with Poor Cognitive Function in Postmenopausal Women in Taiwan. Int J Environ Res Public Health. 2021; 18:2345. 10.3390/ijerph18052345 33673620 PMC7967768

[43] van Asselt KM, Kok HS, Pearson PL, Dubas JS, Peeters PH, Te Velde ER, van Noord PA. Heritability of menopausal age in mothers and daughters. Fertil Steril. 2004; 82:1348–51. 10.1016/j.fertnstert.2004.04.047 15533358

[44] Murabito JM, Yang Q, Fox C, Wilson PW, Cupples LA. Heritability of age at natural menopause in the Framingham Heart Study. J Clin Endocrinol Metab. 2005; 90:3427–30. 10.1210/jc.2005-0181 15769979

[45] van der Plaat DA, Pereira M, Pesce G, Potts JF, Amaral AF, Dharmage SC, Garcia-Aymerich JM, Thompson JR, Gómez Real F, Jarvis DL, Minelli C, Leynaert B, and ALEC Project. Age at menopause and lung function: a Mendelian randomisation study. Eur Respir J. 2019; 54:1802421. 10.1183/13993003.02421-2018 31439684

[46] Hartge P. Genetics of reproductive lifespan. Nat Genet. 2009; 41:637–38. 10.1038/ng0609-637 19471299

[47] Finch CE. The menopause and aging, a comparative perspective. J Steroid Biochem Mol Biol. 2014; 142:132–41. 10.1016/j.jsbmb.2013.03.010 23583565 PMC3773529

[48] Kuh D, Cooper R, Moore A, Richards M, Hardy R. Age at menopause and lifetime cognition: Findings from a British birth cohort study. Neurology. 2018; 90:e1673–81. 10.1212/WNL.0000000000005486 29643079 PMC5952972

[49] Song X, Wu J, Zhou Y, Feng L, Yuan JM, Pan A, Koh WP. Reproductive and hormonal factors and risk of cognitive impairment among Singapore Chinese women. Am J Obstet Gynecol. 2020; 223:410.e1–23. 10.1016/j.ajog.2020.02.032 32112728 PMC7483640

[50] Zhao L, Woody SK, Chhibber A. Estrogen receptor β in Alzheimer’s disease: From mechanisms to therapeutics. Ageing Res Rev. 2015; 24:178–90. 10.1016/j.arr.2015.08.001 26307455 PMC4661108

[51] Genin E, Hannequin D, Wallon D, Sleegers K, Hiltunen M, Combarros O, Bullido MJ, Engelborghs S, De Deyn P, Berr C, Pasquier F, Dubois B, Tognoni G, et al. APOE and Alzheimer disease: a major gene with semi-dominant inheritance. Mol Psychiatry. 2011; 16:903–07. 10.1038/mp.2011.52 21556001 PMC3162068

[52] Choi JI, Han KD, Lee DW, Kim MJ, Shin YJ, Lee HN. Relationship between alcohol consumption and age at menopause: The Korea National Health and Nutrition Examination Survey. Taiwan J Obstet Gynecol. 2017; 56:482–86. 10.1016/j.tjog.2017.05.002 28805605

[53] Zhu D, Chung HF, Pandeya N, Dobson AJ, Cade JE, Greenwood DC, Crawford SL, Avis NE, Gold EB, Mitchell ES, Woods NF, Anderson D, Brown DE, et al. Relationships between intensity, duration, cumulative dose, and timing of smoking with age at menopause: A pooled analysis of individual data from 17 observational studies. PLoS Med. 2018; 15:e1002704. 10.1371/journal.pmed.1002704 30481189 PMC6258514

[54] Ding X, Tang R, Zhu J, He M, Huang H, Lin Z, Zhu J. An Appraisal of the Role of Previously Reported Risk Factors in the Age at Menopause Using Mendelian Randomization. Front Genet. 2020; 11:507. 10.3389/fgene.2020.00507 32547598 PMC7274172

[55] Brinton RD, Chen S, Montoya M, Hsieh D, Minaya J. The estrogen replacement therapy of the Women’s Health Initiative promotes the cellular mechanisms of memory and neuronal survival in neurons vulnerable to Alzheimer’s disease. Maturitas. 2000 (Suppl 2); 34:S35–52. 10.1016/s0378-5122(00)00107-9 10915920

[56] Wnuk A, Przepiórska K, Rzemieniec J, Pietrzak B, Kajta M. Selective Targeting of Non-nuclear Estrogen Receptors with PaPE-1 as a New Treatment Strategy for Alzheimer’s Disease. Neurotox Res. 2020; 38:957–66. 10.1007/s12640-020-00289-8 33025361 PMC7591444

[57] Pompili A, Arnone B, Gasbarri A. Estrogens and memory in physiological and neuropathological conditions. Psychoneuroendocrinology. 2012; 37:1379–96. 10.1016/j.psyneuen.2012.01.007 22309827

[58] Dratva J, Gómez Real F, Schindler C, Ackermann-Liebrich U, Gerbase MW, Probst-Hensch NM, Svanes C, Omenaas ER, Neukirch F, Wjst M, Morabia A, Jarvis D, Leynaert B, Zemp E. Is age at menopause increasing across Europe? Results on age at menopause and determinants from two population-based studies. Menopause. 2009; 16:385–94. 10.1097/gme.0b013e31818aefef 19034049

[59] Brinton RD. Investigative models for determining hormone therapy-induced outcomes in brain: evidence in support of a healthy cell bias of estrogen action. Ann N Y Acad Sci. 2005; 1052:57–74. 10.1196/annals.1347.005 16024751

[60] Shao H, Breitner JC, Whitmer RA, Wang J, Hayden K, Wengreen H, Corcoran C, Tschanz J, Norton M, Munger R, Welsh-Bohmer K, Zandi PP, and Cache County Investigators. Hormone therapy and Alzheimer disease dementia: new findings from the Cache County Study. Neurology. 2012; 79:1846–52. 10.1212/WNL.0b013e318271f823 23100399 PMC3525314

