# Supplementary material for: The role of age at menarche and age at menopause in Alzheimer’s disease: evidence from a bidirectional mendelian randomization study
Source: Aging (Albany NY). 2021 Aug 4;13(15):19722–49. doi: 10.18632/aging.203384 (PMC8386554; doi:10.18632/aging.203384)
Supplement: Supplementary Methods [file aging-13-203384-s001.pdf]

SUPPLEMENTARY METHODS

Supplementary Methods 1. The proportion of variance and f-statistic calculations

The proportion of variance

The proportion of variance (conceptually similar to the R^2) for each single-nucleotide polymorphism (SNP) was calculated using the formula below [1]. The pooled variance of the SNPs was calculated in an additive model assuming no interaction between the individual SNPs.

$$R^2 = \frac{2 \times 2 \times MAF \times (1 - MAF)}{2\beta 2 \times MAF \times (1 - MAF) + (SE(\beta))2 \times 2N \times MAF \times (1 - MAF)}$$

where β is the effect size (beta coefficient) for each SNP; MAF is the minimum allele frequency; SE(β) is the standard error of effect size, and N is the sample size.

F-statistic

The F-statistic of instrument variable was calculated using the formula below [2].

$$F = \frac{\beta^2}{SE(\beta)^2}$$

where β is the effect size (beta coefficient) for each SNP; SE(β) is the standard error of effect size.

Supplementary Methods 2. Details of studies and participants

The reproductive genetics (reprogen) consortium

The ReproGen consortium is an international network of investigators interested in better understanding the genetic basis of reproductive aging. They use large-scale meta-analyses of Genome-wide Association Study (GWAS) data to highlight genetic variants and genes that impact reproductive timing in humans.

Age at menarche

We used the summary data for age at menarche HapMap 2 GWAS meta- analysis results from Perry et al. [3] released by the ReproGen consortium. They meta-analyzed for self-reported age at menarche in a total of 182,416 women of European ancestry from 58 GWAS datasets. Women with self-reported age at menarche of 9-17 years old were included in the analysis, and birth year as the only covariates to allow for the secular trends in menarche timing. The mean age of participants ranged from 15.8 to 79.08 years old, along with the self-reported mean age at menarche ranged from 12.4 to 13.7 years old. Genome-wide SNP array data were available on up to 132,989 women from 57 studies. Each study imputed genotype data based on HapMap Phase II CEU build 35 or 36. SNPs were excluded from individual study datasets if they were poorly imputed or were rare (minor allele frequency, MAF < 1%). Test statistics for each study were adjusted using study-specific genomic control inflation factors and where appropriate individual studies performed additional adjustments for relatedness. Association statistics for each of the 2,441,815 autosomal SNPs that passed quality control (QC) in at least half of the studies were combined across studies in a fixed effects inverse-variance meta-analysis implemented in METAL. On meta-analysis, 3,915 SNPs reached the genome-wide significance threshold ($P<5\times10^{-8}$) for association with age at menarche, and they identified 23 independent signals for age at menarche at 106 genomic loci, and including 11 loci containing multiple independent signals using GCTA. The overall GC inflation factor was 1.266, consistent with an expected high yield of true positive findings in large-scale GWAS meta-analysis of highly polygenic traits.

Age at menopause

We used the summary data for age at menopause HapMap 2 GWAS meta-analysis results from Day et al. [4] released by the ReproGen consortium. They meta-analyzed for self-reported age at natural (non-surgical) menopause (ANM) involving up to 69,360 women of European ancestry from 33 GWAS datasets. Age at menopause was defined as the age at last naturally occurring menstrual period followed by at least 12 consecutive months of amenorrhea. The women with age at natural menopause of 40-60 years old were included, excluding those with menopause induced by hysterectomy, bilateral ovariectomy, radiation or chemotherapy, and those using hormone replacement therapy (HRT) before menopause. Studies were asked to use the full imputed set of HapMap Phase 2 autosomal SNPs, and to run an additive model including top principal components and study specific covariates. SNPs were filtered out if the MAF was less than 1%, or if the imputation quality metrics were low (imputation quality < 0.4). Studies and SNPs passing QC were combined using an inverse-variance weighted meta-analysis, implemented using METAL. Again, this meta-analysis was run by two analysts independently, who then separately used PLINK clumping commands to identify the most significant SNPs in associated regions (termed “Index SNPs”), using only those SNPs which had data from more than 50% of the studies. Finally, they reported 1,208 SNPs reached the genome-wide significance threshold ($P < 5\times10^{-8}$) for association with ANM, and identified independent signals located in 44 genomic regions using approximate conditional analysis implemented in GCTA.

International genomics of Alzheimer's project (IGAP)

We used the largest summary statistics from the 2013 meta-analysis of GWAS data in Alzheimer's disease (AD) released by the IGAP [5]. Details on the design of the arrays, sample processing and QC have been previously described in the original studies. In brief, the IGAP is a large two-stage GWAS study based on individuals of European ancestry. AD cases were confirmed by autopsy- or clinical diagnosis according to National Institute of Neurological and Communicative Disorders and Stroke and the Alzheimer's Disease and Related Disorders Association (NINCDS-ADRDA) criteria, and age, sex and principal components were adjusted for in genetic association analysis. In stage 1, IGAP genotyped and imputed data on 7,055,881 SNPs consisting of 17,008 AD cases and 37,154 controls from four GWAS datasets (the Alzheimer Disease Genetics Consortium [ADGC], the Cohorts for Heart and Aging Research in Genomic Epidemiology consortium [CHARGE], the European Alzheimer's disease Initiative [EADI], and the Genetic and Environmental Risk in AD consortium [GERAD]). The average age of participants was 71 years, with 58.4% were women. In stage 2, 11,632 SNPs were genotyped and tested

for association in an independent set of 8,572 AD cases and 11,312 controls. In our MR study, we only extracted the AD GWAS summary datasets from stage 1 of the IGAP.

The investigators within the IGAP contributed to the design and implementation of IGAP and/or provided data. IGAP was made possible by the generous participation of the control subjects, the patients, and their families. The i–Select chips was funded by the French National Foundation on AD and related disorders. EADI was supported by the LABEX (laboratory of excellence program investment for the future) DISTALZ grant, Inserm, Institut Pasteur de Lille, Université de Lille 2 and the Lille University Hospital. GERAD was supported by the Medical Research Council (Grant n° 503480), Alzheimer's Research UK (Grant n° 503176), the Wellcome Trust (Grant n° 082604/ 2/07/Z) and German Federal Ministry of Education and Research (BMBF): Competence Network Dementia (CND) grant n° 01GI0102, 01GI0711, 01GI0420. CHARGE was partly supported by the NIH/NIA grant R01 AG033193 and the NIA AG081220 and AGES contract N01–AG–12100, the NHLBI grant R01 HL105756, the Icelandic Heart Association, and the Erasmus Medical Center and Erasmus University. ADGC was supported by the NIH/NIA grants: U01 AG032984, U24 AG021886, U01 AG016976, and the Alzheimer's Association grant ADGC–10–196728.

AD-relevant traits

Cognitive performance

We extracted the GWAS summary data of cognitive performance, measured by the respondent's score on a test of verbal cognition, from a sample-size-weighted meta-analysis (N = 257,841) based on healthy individuals of European ancestry performed by Lee JJ et al. [6]. They combined a published study of general cognitive ability (N = 35,298) conducted by the Cognitive Genomics Consortium (COGENT) with new genome-wide association analyses of cognitive performance in the UKB (N = 222,543). The COGENT consortium meta-analyzed 24 cohort studies (comprised of 35 sub-studies) from the general population in North America, the United Kingdom and the European continent. Briefly, each COGENT sub-study administered an average of 8 (SD ± 4) neuropsychological tests. Participant included in COGENT at least had one neuropsychological measure across at least three domains of cognitive performance (for example, digit span for working memory; logical memory for verbal declarative memory; and digit symbol coding for processing speed), or the use of a validated g-sensitive measure was required. Finally, Lee JJ et al. identified 225 genome-wide significant SNPs for cognitive performance.

Genetic investigation of anthropometric traits (GIANT) consortium

We used the largest summary statistics from the 2015 meta-analysis of GWAS data in body mass index (BMI, kg/cm^2) released by GIANT consortium [7]. Briefly, it is a large two-stage GWAS meta-analysis study based on individuals of European ancestry. In stage 1 they performed meta-analysis of 80 GWAS (N = 234,069); and stage 2 incorporated data from 34 additional studies (N = 88,137) genotyped using Metabochip, and adjusted for age, age squared, and any necessary study-specific covariates (for example, genotype-derived principal components) in a linear regression model. Details on the design of the arrays, sample processing and QC have been previously described in the original studies. Finally, this analysis identified 97 BMI-associated loci ($P < 5\times10^{-8}$), accounting for~2.7% of BMI variation, and genome-wide estimates suggest that common variation accounts for >20% of BMI variation.

The tobacco and genetics (TAG) consortium

We used the largest summary statistics from the 2010 meta-analysis of GWAS data for smoking behavior within the cohorts of the TAG consortium, involving up to 74,053 individuals of European ancestry [8]. The TAG consortium conducted GWAS meta-analyses across 16 studies originally designed to evaluate other phenotypes (for example, cardiovascular disease and type 2 diabetes). The 16 TAG studies performed their own genotyping, quality control, and imputation, and study sample size ranged from 585 to 22,307, with the mean age varied from 39.6 to 70.5 years old. In this TAG meta-analysis, four smoking phenotypes-smoking initiation (ever versus never been a regular smoker), age of smoking initiation, smoking quantity (number of cigarettes smoked per day, CPD) and smoking cessation (former versus current smokers) were carefully examined and harmonized. Finally, they performed genotype imputation resulting in a common set of ~2.5 million SNPs, and identified three loci associated with CPD, eight SNPs exceeded genome-wide significance for smoking initiation, and one SNP significantly associated with smoking cessation.

UK biobank (UKB)

We extracted the summary data of self-reported alcohol consumption from a GWAS performed by UKB, comprising of 112 117 white British individuals [9]. UKB is a population-based sample involving 502 629 individuals age of 40 to 69 years resident in the United Kingdom. In this study, participants were asked to report their current drinking status (never, previous, current, prefer not to say) and average weekly and

monthly alcohol consumption of a range of drink types (red wine, white wine, champagne, spirits, beer/cider, fortified wine). After excluding all former drinkers from the analysis, alcohol consumption was derived an average intake of alcohol consumption in units per week (mean = 15.13, SD = 16.56), and was then log (units +1) transformed, this left 112 117 individuals with data on both alcohol consumption and genome-wide genotype data. Consideration of the mean alcohol intake in males was significantly higher than in females, they regressed age and weight in kg onto weekly units of alcohol consumed in males and females separately. Finally, the sample comprised 52.7% of females, with the SNP-based heritability of alcohol consumption in females was estimated to be 13%, and sex-specific analyses found largely overlapping GWAS loci and the genetic correlation between male and female alcohol consumption was 0.90.

Supplementary Methods 3. Sample size and power calculations

We estimated MR power for binary and continuous outcomes at a two-sided α of 0.05, using the mRnd power calculation tool (https://shiny.cnsgenomics.com/mRnd/). MR power calculation given a desired sample size (outcome) relies on the following parameters: the proportion of variance (R^2) explained by genetic instruments in the exposure; the causal effect of the exposure on the outcome, which can be projected across plausible values to investigate impact on statistical power; and the ratio of cases to controls (for binary outcome). While the required sample size for MR given a desired power also relies on several parameters mention above.

The sample size and power calculations for MR analyses are presented in Supplementary Table 1.

REFERENCES

1. Shim H, Chasman DI, Smith JD, Mora S, Ridker PM, Nickerson DA, Krauss RM, Stephens M. A multivariate genome-wide association analysis of 10 LDL subfractions, and their response to statin treatment, in 1868 Caucasians. PLoS One. 2015; 10:e0120758. https://doi.org/10.1371/journal.pone.0120758 PMID:25898129

2. Pierce BL, Ahsan H, Vanderweele TJ. Power and instrument strength requirements for Mendelian randomization studies using multiple genetic variants. Int J Epidemiol. 2011; 40:740–52. https://doi.org/10.1093/ije/dyq151 PMID:20813862

3. Perry JR, Day F, Elks CE, Sulem P, Thompson DJ, Ferreira T, He C, Chasman DI, Esko T, Thorleifsson G, Albrecht E, Ang WQ, Corre T, et al, Australian Ovarian Cancer Study, GENICA Network, kConFab, LifeLines Cohort Study, InterAct Consortium, and Early Growth Genetics (EGG) Consortium. Parent-of-origin-specific allelic associations among 106 genomic loci for age at menarche. Nature. 2014; 514:92–97. https://doi.org/10.1038/nature13545 PMID:25231870

4. Day FR, Ruth KS, Thompson DJ, Lunetta KL, Pervjakova N, Chasman DI, Stolk L, Finucane HK, Sulem P, Bulik-Sullivan B, Esko T, Johnson AD, Elks CE, et al, PRACTICAL consortium, kConFab Investigators, AOCS Investigators, Generation Scotland, EPIC-InterAct Consortium, and LifeLines Cohort Study. Large-scale genomic analyses link reproductive aging to hypothalamic signaling, breast cancer susceptibility and BRCA1-mediated DNA repair. Nat Genet. 2015; 47:1294–303. https://doi.org/10.1038/ng.3412 PMID:26414677

5. Lambert JC, Ibrahim-Verbaas CA, Harold D, Naj AC, Sims R, Bellenguez C, DeStafano AL, Bis JC, Beecham GW, Grenier-Boley B, Russo G, Thorton-Wells TA, Jones N, et al, European Alzheimer's Disease Initiative (EADI), Genetic and Environmental Risk in Alzheimer's Disease, Alzheimer's Disease Genetic Consortium, and Cohorts for Heart and Aging Research in Genomic Epidemiology. Meta-analysis of 74,046 individuals identifies 11 new susceptibility loci for Alzheimer's disease. Nat Genet. 2013; 45:1452–58. https://doi.org/10.1038/ng.2802 PMID:24162737

6. Lee JJ, Wedow R, Okbay A, Kong E, Maghzian O, Zacher M, Nguyen-Viet TA, Bowers P, Sidorenko J, Karlsson Linnér R, Fontana MA, Kundu T, Lee C, et al, 23andMe Research Team, COGENT (Cognitive Genomics Consortium), and Social Science Genetic Association Consortium. Gene discovery and polygenic prediction from a genome-wide association study of educational attainment in 1.1 million individuals. Nat Genet. 2018; 50:1112–21. https://doi.org/10.1038/s41588-018-0147-3 PMID:30038396

7. Genetic studies of body mass index yield new insights for obesity biology. Nature. 2015; 518:197–206. https://doi.org/10.1038/nature14177 PMID:25673413

8. Tobacco and Genetics Consortium. Genome-wide meta-analyses identify multiple loci associated with smoking behavior. Nat Genet. 2010; 42:441–47. https://doi.org/10.1038/ng.571 PMID:20418890

9. Clarke TK, Adams MJ, Davies G, Howard DM, Hall LS, Padmanabhan S, Murray AD, Smith BH, Campbell A, Hayward C, Porteous DJ, Deary IJ, McIntosh AM. Genome-wide association study of alcohol consumption and genetic overlap with other health-related traits in UK Biobank (N=112 117). Mol Psychiatry. 2017; 22:1376–84. https://doi.org/10.1038/mp.2017.153 PMID:28937693

10. Gilsanz P, Lee C, Corrada MM, Kawas CH, Quesenberry CP Jr, Whitmer RA. Reproductive period and risk of dementia in a diverse cohort of health care members. Neurology. 2019; 92:e2005–14. https://doi.org/10.1212/WNL.0000000000007326 PMID:30923235

11. Najar J, Östling S, Waern M, Zettergren A, Kern S, Wetterberg H, Hällström T, Skoog I. Reproductive period and dementia: A 44-year longitudinal population study of Swedish women. Alzheimers Dement. 2020; 16:1153–63. https://doi.org/10.1002/alz.12118 PMID:32573980

12. Kamat MA, Blackshaw JA, Young R, Surendran P, Burgess S, Danesh J, Butterworth AS, Staley JR.

PhenoScanner V2: an expanded tool for searching human genotype-phenotype associations. Bioinformatics. 2019; 35:4851–53. https://doi.org/10.1093/bioinformatics/btz469 PMID:31233103

13. Buniello A, MacArthur JA, Cerezo M, Harris LW, Hayhurst J, Malangone C, McMahon A, Morales J, Mountjoy E, Sollis E, Suveges D, Vrousgou O, Whetzel PL, et al. The NHGRI-EBI GWAS Catalog of published genome-wide association studies, targeted arrays and summary statistics 2019. Nucleic Acids Res. 2019; 47:D1005–12. https://doi.org/10.1093/nar/gky1120 PMID:30445434
