# Supplementary material for: The role of age at menarche and age at menopause in Alzheimer’s disease: evidence from a bidirectional mendelian randomization study
Source: Aging (Albany NY). 2021 Aug 4;13(15):19722–49. doi: 10.18632/aging.203384 (PMC8386554; doi:10.18632/aging.203384)
Supplement: Supplementary Figures [file aging-13-203384-s002.pdf]

SUPPLEMENTARY FIGURES

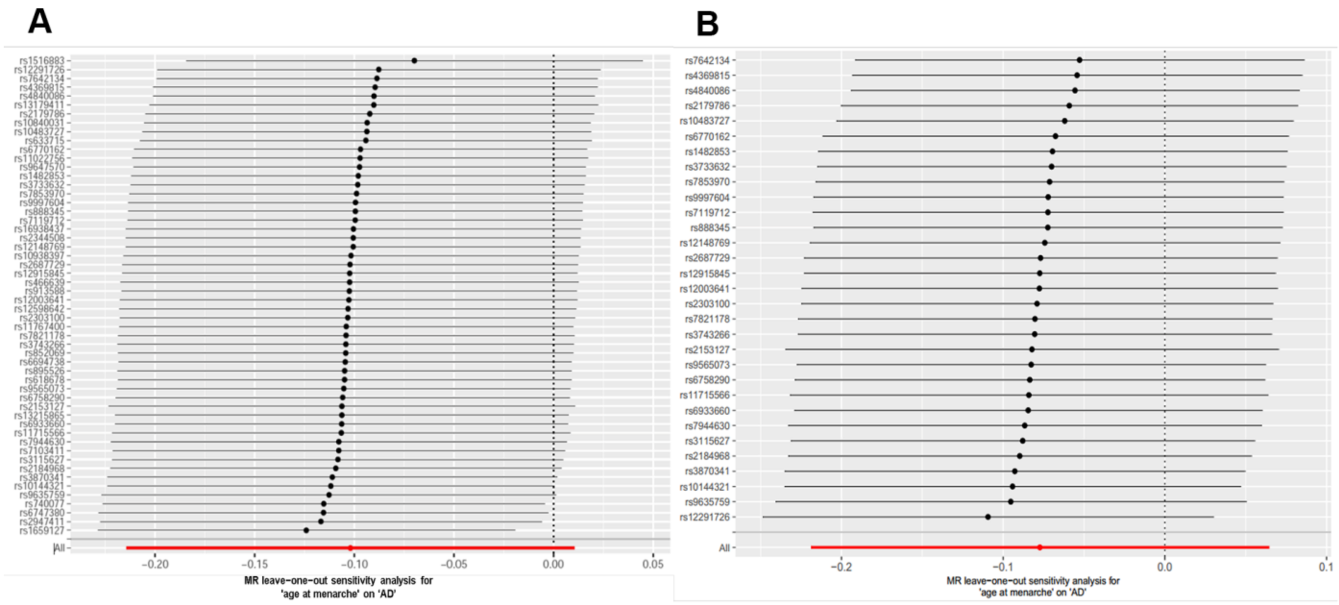

Supplementary Figure 1. MR leave-one-out analysis plots for the relationships of age at menarche with AD. (**A**) MR leave-one-out analysis plots before removing pleiotropic IVs. (**B**) MR leave-one-out analysis plots after removing pleiotropic IVs. Abbreviation: MR, mendelian randomization; AD, Alzheimer's disease; IVs, instrument variables.

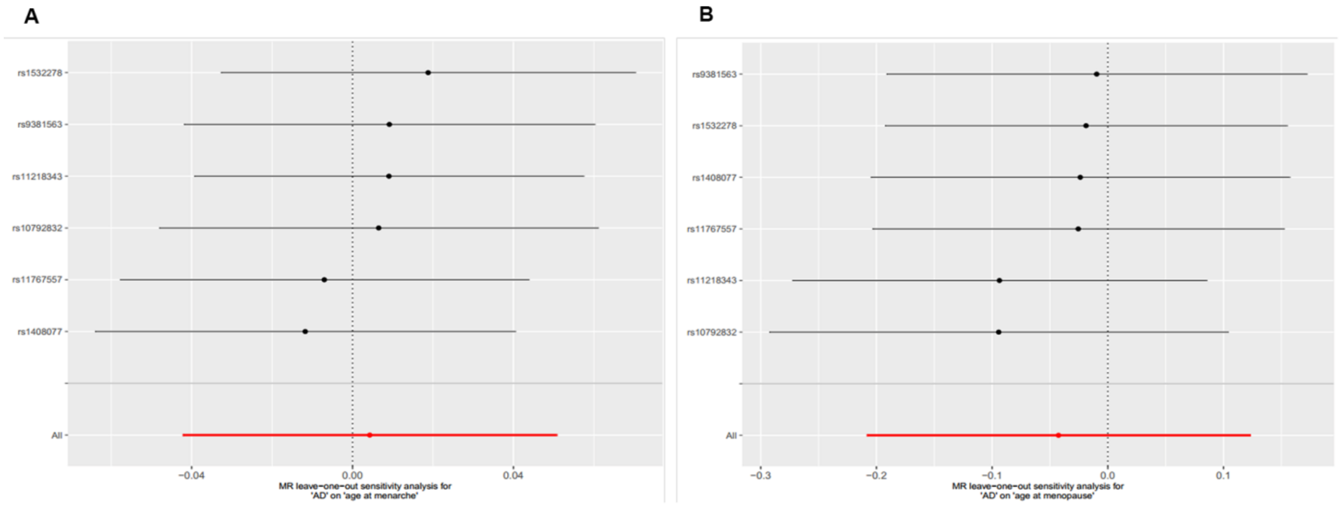

Supplementary Figure 2. MR leave-one-out analysis plots for the relationships of AD with age at menarche/menopause. (**A**) MR leave-one-out analysis plot for the relationships of AD with age at menarche. (**B**) MR leave-one-out analysis plot for the relationships of AD with age at menopause. Abbreviation: MR, mendelian randomization; AD, Alzheimer's disease.

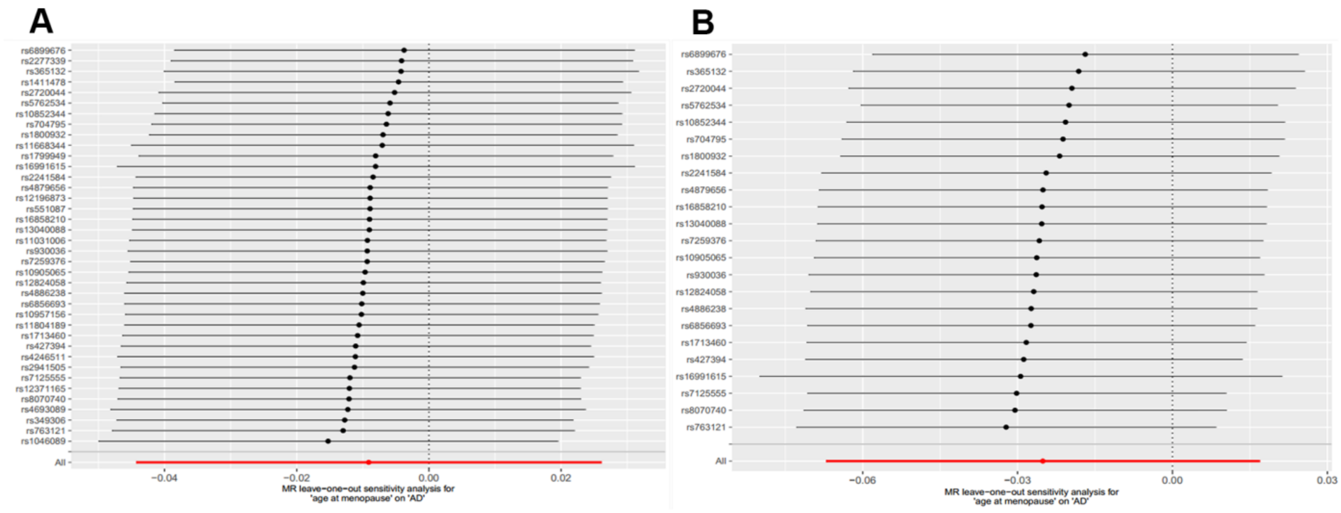

Supplementary Figure 3. MR leave-one-out analysis plots for the relationships of age at menopause with AD. (**A**) MR leave-one-out analysis plots before removing pleiotropic IVs. (**B**) MR leave-one-out analyses plots after removing pleiotropic IVs. Abbreviation: MR, mendelian randomization; AD, Alzheimer's disease; IVs, instrument variables.
