# Supplementary material for: The role of age at menarche and age at menopause in Alzheimer’s disease: evidence from a bidirectional mendelian randomization study
Source: Aging (Albany NY). 2021 Aug 4;13(15):19722–49. doi: 10.18632/aging.203384 (PMC8386554; doi:10.18632/aging.203384)
Supplement: Supplementary Tables [file aging-13-203384-s003.pdf]

SUPPLEMENTARY TABLES

Supplementary Table 1. The sample size and power calculations for MR analyses (two-sided α=0.05).

Exposure-outcome	Actual N (outcome-GWAS)	Ratio of cases to controls (outcome-GWAS)	Observational HR	$R^2$of IVs (%)	N required for 80% power	Power at actual N (%)
**Age at menarche/menopause-AD (binary)**						
Age at menarche-AD	54,162	0.458	1.23[10]	3.55	24,000	98.8
Age at menopause-AD	54,162	0.458	1.17[11]	4.69	31,600	95.7
**AD-age at menarche/menopause (continuous)**						
Exposure-outcome	Actual N (outcome-GWAS)	Ratio of cases to controls (outcome-GWAS)	Observational β*	$R^2$of IVs (%)	N required for 80% power	Power at actual N (%)
AD-age at menarche	182,416	/	0.21[10]	3.37	5,500	100
AD-age at menopause	69,360	/	0.16[11]	3.37	9,500	100

Abbreviations: AD, Alzheimer's disease; GWAS, genome-wide association study; IVs, instrument variables; HR, hazard ratio.
*β equals to ln (HR).

Supplementary Table 2. Genome-wide significant SNPs (n = 68) for age at menarche ($P < 1×10^{-8}$).

SNP ID [a]	Proxy SNP [b]	r^2 for proxy	Effect allele (alternative)	Beta (SE) for age at menarche	Beta (SE) for AD	Variance explained (R^2)	F statistic
rs10144321	-	-	G (A)	-0.042 (0.007)	-0.023 (0.018)	0.0006	36
rs10483727	-	-	C (T)	-0.037 (0.006)	0.026 (0.017)	0.0006	38
rs1079866	-	-	G (C)	0.072 (0.008)	-0.009 (0.023)	0.0011	81
rs10840031	-	-	A (G)	0.038 (0.006)	-0.032 (0.019)	0.0005	40
rs10938397	-	-	G (A)	-0.038 (0.006)	0.005 (0.016)	0.0007	40
rs11022756	-	-	C (A)	-0.048 (0.006)	0.016 (0.017)	0.0009	64
rs11715566	-	-	T (C)	0.052 (0.006)	0.002 (0.015)	0.0014	75
rs11756454	rs2249703	0.83	A (T)	0.034 (0.006)	0.001 (0.015)	0.0006	32
rs11767400	-	-	A (C)	0.035 (0.006)	0.003 (0.017)	0.0005	34
rs12003641	-	-	T (C)	0.082 (0.011)	-0.006 (0.028)	0.0009	56
rs12148769	-	-	A (G)	-0.055 (0.010)	0.012 (0.026)	0.0006	30
rs12291726	-	-	G (A)	-0.057 (0.008)	-0.046 (0.021)	0.0006	51
rs12598642	-	-	G (A)	0.044 (0.006)	-0.021 (0.016)	0.0010	54
rs12915845	-	-	T (C)	-0.035 (0.006)	0.003 (0.016)	0.0006	34
rs13179411	-	-	T (G)	0.060 (0.008)	-0.038 (0.021)	0.0010	56
rs13215865	-	-	T (C)	-0.042 (0.007)	-0.010 (0.020)	0.0004	36
rs1398217	-	-	C (G)	0.046 (0.006)	0.009 (0.016)	0.0010	59
rs1482853	-	-	A (C)	-0.038 (0.006)	0.013 (0.016)	0.0007	40
rs1516883	-	-	A (G)	-0.091 (0.002)	0.042 (0.017)	0.0035	2070
rs1518080	-	-	G (C)	-0.051 (0.006)	0.045 (0.016)	0.0013	72
rs1659127	-	-	A (G)	0.044 (0.006)	0.052 (0.018)	0.0008	54
rs16938437	-	-	T (C)	-0.067 (0.010)	0.013 (0.028)	0.0004	45
rs17351680	-	-	G (C)	0.044 (0.008)	-0.004 (0.022)	0.0004	30
rs1874984	-	-	C (G)	0.037 (0.006)	0.007 (0.018)	0.0007	38
rs2153127	-	-	C (T)	-0.077 (0.002)	0.003 (0.016)	0.0029	1482
rs2179786	-	-	T (G)	-0.039 (0.006)	0.026 (0.016)	0.0007	42
rs2184968	-	-	C (T)	-0.036 (0.006)	-0.014 (0.016)	0.0006	36
rs2303100	-	-	T (C)	0.038 (0.006)	-0.000 (0.017)	0.0007	40
rs2344508	-	-	A (G)	0.034 (0.006)	-0.007 (0.016)	0.0006	32
rs2617056	-	-	T (A)	-0.036 (0.006)	-0.003 (0.016)	0.0006	36
rs2687729	-	-	G (A)	0.044 (0.007)	-0.004 (0.017)	0.0007	40

rs2836950	-	-	G (C)	-0.035 (0.006)	-0.018 (0.017)	0.0005	34
rs2947411	-	-	G (A)	-0.052 (0.008)	-0.034 (0.020)	0.0006	42
rs3115627	-	-	G (A)	0.038 (0.006)	0.014 (0.018)	0.0007	40
rs3733632	-	-	G (A)	0.049 (0.008)	-0.017 (0.021)	0.0009	38
rs3743266	-	-	C (T)	-0.045 (0.006)	-0.001 (0.017)	0.0009	56
rs3870341	-	-	G (A)	-0.043 (0.006)	-0.019 (0.018)	0.0008	51
rs3914188	-	-	C (G)	0.044 (0.007)	-0.013 (0.018)	0.0007	40
rs4242496	-	-	A (T)	-0.033 (0.006)	0.005 (0.016)	0.0005	30
rs4369815	-	-	G (T)	-0.080 (0.012)	0.064 (0.032)	0.0006	44
rs466639	-	-	C (T)	0.075 (0.008)	-0.007 (0.025)	0.0013	88
rs4801589	-	-	G (C)	0.032 (0.006)	-0.004 (0.017)	0.0005	28
rs4840086	rs2894891	1.00	G (A)	-0.036 (0.006)	0.034 (0.016)	0.0006	36
rs618678	-	-	T (C)	-0.034 (0.006)	-0.006 (0.017)	0.0005	32
rs633715	-	-	C (T)	-0.051 (0.007)	0.028 (0.021)	0.0008	53
rs6694738	rs7522883	0.98	A (C)	-0.044 (0.008)	0.011 (0.027)	0.0006	30
rs6747380	-	-	A (G)	0.065 (0.008)	0.025 (0.021)	0.0012	66
rs6758290	-	-	C (T)	-0.040 (0.006)	-0.006 (0.017)	0.0008	44
rs6770162	-	-	A (G)	0.036 (0.006)	-0.017 (0.016)	0.0006	36
rs6933660	-	-	A (C)	-0.036 (0.006)	-0.008 (0.017)	0.0005	36
rs7103411	-	-	T (C)	-0.043 (0.007)	-0.012 (0.019)	0.0006	38
rs7119712	-	-	A (G)	-0.041 (0.006)	0.011 (0.018)	0.0005	47
rs740077	-	-	C (A)	-0.046 (0.007)	-0.031 (0.019)	0.0007	43
rs7642134	-	-	G (A)	0.038 (0.006)	-0.036 (0.016)	0.0007	40
rs7821178	-	-	A (C)	-0.045 (0.006)	-0.000 (0.017)	0.0010	56
rs7853970	-	-	C (T)	-0.037 (0.006)	0.013 (0.018)	0.0007	38
rs7944630	-	-	A (G)	0.047 (0.006)	0.006 (0.016)	0.0011	61
rs852069	-	-	G (A)	0.036 (0.006)	0.002 (0.016)	0.0006	36
rs888345	-	-	A (G)	-0.044 (0.007)	0.014 (0.022)	0.0006	40
rs895526	-	-	C (T)	0.044 (0.008)	0.006 (0.021)	0.0006	30
rs913588	-	-	A (G)	-0.034 (0.006)	0.002 (0.015)	0.0006	32
rs9373571	-	-	A (T)	0.034 (0.006)	0.009 (0.016)	0.0006	32
rs9555810	-	-	G (C)	0.047 (0.006)	-0.013 (0.018)	0.0009	61
rs9565073	-	-	C (T)	0.034 (0.006)	0.006 (0.016)	0.0006	32
rs9635759	-	-	A (G)	0.058 (0.006)	0.013 (0.017)	0.0015	93
rs9647570	-	-	G (T)	0.046 (0.008)	-0.023 (0.023)	0.0004	33
rs9939609	-	-	A (T)	-0.042 (0.005)	0.006 (0.016)	0.0009	71
rs9997604	-	-	C (A)	0.039 (0.007)	-0.011 (0.017)	0.0006	31

Abbreviations: AD, Alzheimer's disease; SNP, single-nucleotide polymorphism; SE, Standard error.

[a]Fourteen SNPs (rs1079866, rs11756454, rs1398217, rs1518080, rs17351680, rs1874984, rs2617056, rs2836950, rs3914188, rs4242496, rs4801589, rs9373571, rs9555810, rs9939609) being palindromic were removed, and 54 SNPs were included for MR analyses.

[b]Proxy SNP reported where the targeted SNP was not available in the outcome datasets, and the effect allele and beta (SE) reported for proxy SNP.

Supplementary Table 3. Genome-wide significant SNPs (n = 42) for age at menopause ($P < 1×10^{-8}$).

SNP ID [a]	Proxy SNP [b]	r^2 for proxy	Effect allele (alternative)	Beta (SE) for age at menopause	Beta (SE) for AD	Variance explained (R^2)	F statistic
rs1046089	-	-	A (G)	-0.220 (0.020)	-0.034 (0.017)	0.0211	121
rs1054875	-	-	T (A)	-0.190 (0.020)	-0.009 (0.016)	0.0170	90
rs10852344	-	-	T (C)	-0.160 (0.020)	0.025 (0.017)	0.0128	64
rs10905065	-	-	A (G)	-0.110 (0.020)	-0.005 (0.016)	0.0058	30
rs10957156	-	-	A (G)	-0.140 (0.020)	-0.010 (0.018)	0.0067	49
rs11031006	-	-	A (G)	0.220 (0.030)	0.000 (0.022)	0.0095	54
rs11668344	-	-	G (A)	-0.410 (0.020)	0.010 (0.017)	0.0765	420
rs11804189	-	-	A (G)	0.110 (0.020)	0.015 (0.016)	0.0059	30
rs12196873	-	-	C (A)	0.160 (0.030)	-0.005 (0.023)	0.0050	28
rs12371165	-	-	T (C)	0.180 (0.030)	0.038 (0.023)	0.0091	36
rs12599106	-	-	A (T)	-0.120 (0.020)	-0.022 (0.022)	0.0072	36
rs12824058	-	-	G (A)	-0.140 (0.020)	-0.006 (0.017)	0.0095	49
rs13040088	-	-	G (A)	-0.160 (0.020)	0.003 (0.020)	0.0069	64
rs1411478	-	-	G (A)	0.130 (0.020)	-0.039 (0.016)	0.0082	42
rs16858210	-	-	A (G)	0.140 (0.020)	-0.003 (0.018)	0.0067	49
rs16991615	-	-	A (G)	0.880 (0.040)	-0.013 (0.032)	0.1140	484
rs1713460	-	-	G (A)	-0.140 (0.020)	-0.015 (0.017)	0.0069	49
rs1799949	-	-	A (G)	0.140 (0.020)	-0.010 (0.016)	0.0089	49
rs1800932	-	-	G (A)	0.170 (0.030)	-0.028 (0.022)	0.0102	32
rs2236918	-	-	G (C)	0.150 (0.020)	0.009 (0.016)	0.0111	56
rs2241584	-	-	A (G)	-0.140 (0.020)	0.007 (0.016)	0.0095	49
rs2277339	-	-	G (T)	-0.310 (0.030)	0.054 (0.027)	0.0188	107
rs2720044	-	-	C (A)	0.290 (0.030)	-0.032 (0.022)	0.0237	93
rs2941505	-	-	G (A)	0.130 (0.020)	0.019 (0.017)	0.0070	42
rs349306	-	-	A (G)	0.230 (0.040)	0.055 (0.029)	0.0112	33
rs365132	-	-	T (G)	0.240 (0.020)	-0.024 (0.016)	0.0122	144
rs4246511	-	-	C (T)	-0.220 (0.020)	-0.012 (0.019)	0.0199	121
rs427394	-	-	G (A)	-0.130 (0.020)	-0.016 (0.016)	0.0082	42
rs4693089	-	-	G (A)	0.200 (0.020)	0.015 (0.016)	0.0199	100
rs4879656	-	-	A (C)	-0.120 (0.020)	0.003 (0.016)	0.0072	36
rs4886238	-	-	A (G)	0.180 (0.020)	0.004 (0.016)	0.0151	81
rs551087	-	-	A (G)	0.130 (0.020)	-0.003 (0.017)	0.0054	42
rs5762534	-	-	C (T)	0.160 (0.030)	-0.047 (0.022)	0.0079	28
rs6856693	-	-	G (A)	0.160 (0.020)	0.007 (0.017)	0.0127	64
rs6899676	-	-	G (A)	0.230 (0.030)	-0.041 (0.020)	0.0149	59
rs704795	-	-	A (G)	-0.160 (0.020)	0.021 (0.016)	0.0127	64
rs7125555	-	-	T (C)	-0.120 (0.020)	-0.023 (0.016)	0.0072	36
rs7259376	-	-	G (A)	0.110 (0.020)	0.001 (0.016)	0.0060	30
rs763121	-	-	G (A)	-0.160 (0.020)	-0.027 (0.016)	0.0118	64
rs8070740	-	-	G (A)	0.150 (0.020)	0.028 (0.018)	0.0054	56
rs930036	-	-	A (G)	-0.190 (0.020)	0.001 (0.016)	0.0170	90
rs9796	-	-	T (A)	-0.130 (0.020)	-0.002 (0.016)	0.0079	42

Abbreviations: AD, Alzheimer's disease; SNP, single-nucleotide polymorphism; SE, Standard error.

[a]Four SNPs (rs1054875, rs12599106, rs2236918, rs9796) being palindromic were removed, and 38 instrument SNPs were included for MR analyses.

[b]Proxy SNP reported where the targeted SNP was not available in the outcome datasets, and the effect allele and beta (SE) reported for proxy SNP.

Supplementary Table 4. Genome-wide significant SNPs (n = 17) for AD ($P < 1×10^{-8}$).

SNP ID [a]	Proxy SNP [b]	r^2 for proxy	Effect allele (alternative)	AD (exposure)-age at menarche(outcome)		AD (exposure)-age at menopause (outcome)		Variance explained (R^2)	F statistic
				Beta (SE) for AD	Beta (SE) for age at menarche	Beta (SE) for AD	Beta (SE) for age at menopause		
rs10792832	-	-	G (A)	0.130 (0.016)	0.001 (0.007)	0.130 (0.016)	0.010 (0.020)	0.0079	65
rs10808026	rs11767557	0.97	C (T)	-0.129 (0.021)	0.008 (0.008)	-0.139 (0.021)	0.020 (0.030)	0.0058	39
rs11218343	-	-	C (T)	-0.270 (0.041)	-0.014 (0.023)	-0.270 (0.041)	-0.070 (0.060)	0.0060	43
rs118170342	-	-	C (T)	0.871 (0.057)	-	0.871 (0.057)	-	0.0594	233
rs12590654	-	-	A (G)	-0.097 (0.018)	-	-0.097 (0.018)	-	0.0042	30
rs1752684	rs1408077	0.9335	A (C)	-0.143 (0.020)	0.009 (0.007)	-0.154 (0.020)	0.020 (0.030)	0.0060	53
rs346771	-	-	C (T)	0.303 (0.040)	-	0.303 (0.040)	-	0.0134	58
rs41289512	-	-	G (C)	1.638 (0.059)	-	1.638 (0.059)	-	0.1602	760
rs41290100	-	-	T (C)	-0.570 (0.103)	-	-0.570 (0.103)	-	0.0163	31
rs41290120	-	-	A (G)	-0.608 (0.050)	-	-0.608 (0.050)	-	0.0214	146
rs4147929	-	-	G (A)	-0.135 (0.022)	-	-0.135 (0.022)	-	0.0055	36
rs4663105	-	-	C (A)	0.184 (0.017)	-	0.184 (0.017)	-	0.0163	114
rs72924659	-	-	T (C)	-0.141 (0.020)	-	-0.141 (0.020)	-	0.0083	52
rs7982	rs1532278	0.98	T (C)	0.143 (0.017)	0.009 (0.008)	0.140 (0.017)	-0.040 (0.040)	0.0097	75
rs8093731	-	-	T (C)	-0.614 (0.112)	-	-0.614 (0.112)	-	0.0089	30
rs9272561	-	-	A (G)	-0.136 (0.023)	-	-0.136 (0.023)	-	0.0091	35
rs9381563	-	-	T (C)	-0.097(0.017)	0.002 (0.006)	-0.097(0.017)	-0.020 (0.020)	0.0041	34

Abbreviations: AD, Alzheimer's disease; SNP, single-nucleotide polymorphism; SE, Standard error.

[a]None of SNP was removed for being palindromic, but only 6 IVs were found in outcome (age at menarche/ menopause) datasets and included for MR analyses.

[b]Proxy SNP reported where the targeted SNP was not available in the outcome datasets, and the effect allele and beta (SE) reported for proxy SNP.

Supplementary Table 5. GWAS linked traits of 54 instrument SNPs of age at menarche.

SNP ID	Phenoscanner [12]	dbSNP genes	GWAS catalog [13] traits linked to this gene
rs10144321	Age at menarche	WDR25	Age at menarche, height
rs10483727	Height, arm fat-free mass right	NA	NA
rs10840031*	BMI	STK33	BMI
rs10938397*	BMI, obesity	NA	NA
rs11022756*	BMI, coronary artery disease	NA	NA
rs11715566	Relative age voice broke	LOC107986022	NA
rs11767400*	Height	CADPS2	BMI
rs12003641	Height, Relative age voice broke	NA	NA
rs12148769	Age at menarche	NA	NA
rs12291726	Impedance of arm left	GAB2	eGFR, AD, TG, TC
rs12598642*	BMI, trunk fat mass, self-reported diabetes	WWP2	IgE levels, smoking behavior
rs12915845	Creatinine in urine, relative age voice broke	NA	NA
rs13179411*	BMI, relative age voice broke	JADE2	BMI, T2DM, mental health
rs13215865*	Age at menarche	JADE2	BMI, T2DM, mental health
rs1482853	Birth weight, WC	LINC02029	NA
rs1516883*	BMI, WC adjusted for smoking	NA	NA
rs1659127*	Leg fat-free mass right	NA	NA
rs16938437*	Arm fat-free mass left, weight	PHF21A	BMI, educational attainment, smoking initiation
rs2153127	Relative age voice broke	NA	NA
rs2179786	Age at menarche	FAM83B	Wellbeing, sleep duration, colorectal cancer
rs2184968	Arm predicted mass right, T2DM, neutrophil count	CENPW	Brain volume measurement, cortical surface area measurement
rs2303100	Sleep duration	OLFM2	Waist-hip ratio, sleep duration
rs2344508*	BMI, hip circumference	TNNI3K	BMI, obesity, smoking initiation
rs2687729	Asthma	EEFSEC	prostate carcinoma
rs2947411*	BMI, WC, leg fat mass right	NA	NA
rs3115627	Rheumatoid arthritis, MS, myeloid white cell count	LOC105375010	NA

rs3733632	Impedance of arm left, height	TACR3	Heel bone mineral density, adolescent idiopathic scoliosis
rs3743266	Relative age voice broke, height	RORA/RORA-AS1	NA
rs3870341	Impedance of whole body	NA	NA
rs4369815	Age at menarche	NA	NA
rs466639*	Age at menarche	RXRG	Bipolar disorder, BMI-adjusted WC, AIDS
rs4840086	Age at menarche	NA	NA
rs618678*	Years of educational attainment, maternal smoking around birth	KDM4A	Educational attainment, squamous cell lung carcinoma, schizophrenia, smoking status
rs633715*	BMI, WC	NA	NA
rs6694738*	Age at menarche	AKT3	BMI, schizophrenia, educational attainment
rs6747380*	Age at menarche	CCDC85A	BMI, colorectal adenoma, self-reported educational attainment
rs6758290	Age at menarche	NA	NA
rs6770162	Age at menarche	NA	NA
rs6933660	Self-reported endometriosis	NA	NA
rs7103411*	BMI, WC	BDNF/BDNF-AS	BMI, coronary artery disease, smoking behavior
rs7119712	Acute sinusitis	TRPC6	Colorectal cancer or advanced adenoma, sleep duration, lung adenocarcinoma
rs740077*	Impedance of whole body, weight	KDM3B	Bipolar disorder, autism spectrum disorder or schizophrenia, sleep duration
rs7642134	Age at menarche	NA	NA
rs7821178	NA	NA	NA
rs7853970	Impedance of leg right	NA	NA
rs7944630	Relative age voice broke, height	NA	NA
rs852069*	BMI	LOC105372544	NA
rs888345	Age at menarche, height	KCNK9	parental longevity
rs895526*	Schizophrenia	SATB2	Intelligence, schizophrenia, educational attainment, general cognitive ability
rs913588*	Schizophrenia, relative age voice broke	KDM4C	Bipolar disorder and schizophrenia, BMI, educational attainment
rs9565073	Age at menarche	KLF12	Total PHF-tau, QRS duration, heel bone mineral density
rs9635759	Age at menarche	NA	NA
rs9647570*	Age at menarche	TENM2/LOC105377709	Smoking status, educational attainment, alcohol consumption, BMI, depression
rs9997604	Age at menarche	NA	NA

Abbreviations: GWAS, genome-wide association study; SNP, single-nucleotide polymorphism; BMI, body mass index; WC, waist circumference; AD, Alzheimer's disease; T2DM, Type 2 diabetes mellitus; TC, total cholesterol; TG, triglyceride; MS, multiple sclerosis; eGFR, glomerular filtration rate; AIDS, acquired immune deficiency syndrome.
*Indicates instrument SNP with potential pleiotropic and was removed in the final MR analyses.

Supplementary Table 6. GWAS linked traits of 6 instrument SNPs of AD.

SNP ID	Phenoscanner [12]	dbSNP genes	GWAS catalog [13] traits linked to this gene
rs10792832	AD in APOE e4 carriers	NA	NA
rs10808026	AD in APOE e5 carriers	EPHA1	AD, blood protein levels
rs11218343	AD in APOE e6 carriers	SORL1	AD, alcohol consumption, insomnia
rs1752684	AD in APOE e7 carriers	CR1	AD, inflammatory biomarkers
rs7982	AD in APOE e8 carriers	CLU	AD, panic disorder, refractive error
rs9381563	Height, reticulocyte count	NA	NA

Abbreviations: GWAS, genome-wide association study; AD, Alzheimer's disease; SNP, single-nucleotide polymorphism.

Supplementary Table 7. GWAS linked traits of 38 instrument SNPs of age at menopause.

SNP ID	Phenoscanner [12]	dbSNP genes	GWAS catalog [13] traits linked to this gene
rs1046089*	T1DM, white blood cell count, schizophrenia	PRRC2A	BMI, WC, schizophrenia, smoking status
rs10852344	Menopause age at onset	NA	NA
rs10905065	Age at menopause	TASOR2	Osteosarcoma, breast cance, cutaneous malignant melanoma
rs10957156*	Neutrophil percentage of granulocytes	CHD7	Smoking initiation, MDD
rs11031006*	Polycystic ovary syndrome	CHD7	Smoking initiation, MDD
rs11668344*	Ever used hormone-replacement therapy	CHD7	Smoking initiation, MDD
rs11804189*	Age at menopause	CHD7	Smoking initiation, MDD
rs12196873*	Age at menopause	MFSD4B	Smoking initiation, T2DM
rs12371165*	Age at menopause	GRIP1	Basophil percentage of white cells, PHF-tau measurement
rs12824058	Age at menopause	NA	NA
rs13040088	Age at menopause	DIDO1	Fat-free mass, monocyte count
rs1411478*	Ever used hormone-replacement therapy	STX6	Creutzfeldt-Jakob disease, progressive supranuclear palsy
rs16858210	Pulse rate	NA	NA
rs16991615	Age at menopause	MCM8	Uterine fibroids, breast cancer
rs1713460	Age at menopause	NA	NA
rs1799949*	Age at menopause	BRCA1	Ovarian cancer, BMI
rs1800932	Ever smoked	MSH6	Heel bone mineral density, tea consumption
rs2241584	Age at menopause	RNF44	Venous thromboembolism
rs2277339*	Platelet crit, height	PRIM1/HSD17B6	Smoking initiation, Mean corpuscular volume, T2DM
rs2720044	Age at menopause	ASH2L	Menopause (age at onset)
rs2941505*	Asthma, HDL, sum basophil neutrophil counts	PGAP3	Lifetime smoking index,TG, bipolar disorder
rs349306*	Age at menopause	ARID3A	Vertical cup-disc ratio, systemic lupus erythematosus
rs365132	Leiomyoma of uterus	UIMC1	Educational attainment, WC adjusted BMI
rs4246511*	Age at menopause	RHBDL2/LOC105378662	TG, MDD, alcohol dependence
rs427394	Age at menopause	TENT4A	MS, Coronary artery disease
rs4693089*	Age at menopause	HELQ	Age-related cognitive decline, oral cavity and pharyngeal cancer
rs4879656	Age at menopause	APTX	Vitamin B12 levels, IgG glycosylation, amyotrophic lateral sclerosis
rs4886238	Age at menopause	TDRD3	Metabolite levels
rs551087*	Age at menopause	SPPL3	T2DM, depression, cognitive performance, educational attainment
rs5762534	Age at menopause	TTC28	Breast cancer, epithelial ovarian cancer, prostate cancer
rs6856693	Age at menopause	ACSL1/ LOC105377587	T2DM, fulminant T1DM
rs6899676	Age at menopause	SYCP2L	COPD
rs704795	TG, serum urate, platelet count, TC	FNDC4	Age at menopause
rs7125555	Age at menopause	NA	NA
rs7259376	Age at menopause	NA	NA
rs763121	TG, mean corpuscular volume	DDX17/KDELR3	TPE interval, gallstone disease
rs8070740	Age at menopause	RPAIN	Neutrophil count, WBC
rs930036	Basal metabolic rate	TLK1	Height, platelet count, self-reported math ability

Abbreviations: GWAS, genome-wide association study; SNP, single-nucleotide polymorphism; BMI, body mass index; WC, waist circumference; T1DM, Type 1 diabetes mellitus; T2DM, Type 2 diabetes mellitus; TC, total cholesterol; TG, triglyceride; HDL, high-density lipoprotein; MS, multiple sclerosis; MDD, major depressive disorder;COPD, chronic obstructive pulmonary disease; WBC, white blood cell count.

*Indicates instrument SNP with potential pleiotropic and was removed in the final MR analyses.

Supplementary Table 8. The heterogeneity and sensitivity results of age at menarche/menopause and AD relevant traits.

Exposure-outcome	No. of SNPs*	MR-PRESSO	MR Egger intercept		Cochran's heterogeneity test			
		Global *P* value	Intercept value	*P* value	IVW-Q value	IVW-*P* value	Egger-Q value	Egger-*P* value
Age at menarche-cognitive performance	41	0.081	0.001	0.588	53.595	0.074	53.189	0.064
Age at menarche-BMI	31	0.306	0.001	0.801	33.567	0.299	33.491	0.258
Age at menarche-smoking behavior	48	0.709	-0.004	0.934	40.716	0.729	40.709	0.693
Age at menarche-alcohol consumption	50	0.451	0.002	0.224	48.867	0.478	47.347	0.499
Age at menopause-cognitive performance	29	0.155	0.000	0.751	35.817	0.147	35.681	0.122
Age at menopause-BMI	36	0.401	-0.002	0.273	36.693	0.390	35.403	0.402
Age at menopause-smoking behavior	30	0.754	0.027	0.519	23.734	0.742	23.306	0.718
Age at menopause-alcohol consumption	31	0.988	0.000	0.784	15.607	0.986	15.530	0.980

Abbreviations: MR, mendelian randomization; AD, Alzheimer's disease; IVW, inverse variance-weighted; BMI, body mass index; SNP, single-nucleotide polymorphism.
*Indicates model removal of potential pleiotropic instrument SNPs.
